# The Interaction of CtIP and Nbs1 Connects CDK and ATM to Regulate HR–Mediated Double-Strand Break Repair

**DOI:** 10.1371/journal.pgen.1003277

**Published:** 2013-02-28

**Authors:** Hailong Wang, Linda Z. Shi, Catherine C. L. Wong, Xuemei Han, Patty Yi-Hwa Hwang, Lan N. Truong, Qingyuan Zhu, Zhengping Shao, David J. Chen, Michael W. Berns, John R. Yates, Longchuan Chen, Xiaohua Wu

**Affiliations:** 1 Department of Molecular and Experimental Medicine, The Scripps Research Institute, La Jolla, California, United States of America; 2 The Institute of Engineering in Medicine, University of California San Diego, La Jolla, California, United States of America; 3 Department of Chemical Physiology, The Scripps Research Institute, La Jolla, California, United States of America; 4 Division of Molecular Radiation Biology, Department of Radiation Oncology, University of Texas Southwestern Medical Center, Dallas, Texas, United States of America; 5 Department of Pathology, Veterans Affairs Medical Center, Long Beach, California, United States of America; National Cancer Institute, United States of America

## Abstract

CtIP plays an important role in homologous recombination (HR)–mediated DNA double-stranded break (DSB) repair and interacts with Nbs1 and BRCA1, which are linked to Nijmegen breakage syndrome (*NBS*) and familial breast cancer, respectively. We identified new CDK phosphorylation sites on CtIP and found that phosphorylation of these newly identified CDK sites induces association of CtIP with the N-terminus FHA and BRCT domains of Nbs1. We further showed that these CDK-dependent phosphorylation events are a prerequisite for ATM to phosphorylate CtIP upon DNA damage, which is important for end resection to activate HR by promoting recruitment of BLM and Exo1 to DSBs. Most notably, this CDK-dependent CtIP and Nbs1 interaction facilitates ATM to phosphorylate CtIP in a substrate-specific manner. These studies reveal one important mechanism to regulate cell-cycle-dependent activation of HR upon DNA damage by coupling CDK- and ATM-mediated phosphorylation of CtIP through modulating the interaction of CtIP with Nbs1, which significantly helps to understand how DSB repair is regulated in mammalian cells to maintain genome stability.

## Author Summary

DNA double-strand break (DSB) repair plays a critical role in the maintenance of genome stability, and mutations in key regulators in the DSB repair pathway are often associated with cancers and human diseases exhibiting chromosomal aberrations. CtIP is an essential DNA repair factor participating in homologous recombination (HR)–mediated DSB repair. In this study, we found that phosphorylation of CtIP by cell-cycle-dependent kinases CDKs modulates CtIP interaction with Nbs1, a component of the Mre11-Rad50-Nbs1 complex that is associated with Nijmegen breakage syndrome (NBS). We also showed that CtIP is phosphorylated by ataxia-telangiectasia gene product ATM, a kinase critical for the activation of DNA damage response pathway. Importantly, we found that CDK-mediated phosphorylation of CtIP is a prerequisite for CtIP to be phosphorylated by ATM upon DNA damage. These findings reveal a novel mechanism of how cell-cycle-dependent activities regulate HR in response to DNA damage, which significantly helps in understanding the regulation of DSB repair in mammalian cells. This study also provides new insight as to how the DNA damage checkpoint and repair proteins that are associated with human diseases participate in the maintenance of genome stability to prevent cancer.

## Introduction

DNA double-strand breaks (DSBs) are often generated during normal cellular processes such as the development of immune systems and meiotic recombination, and they can also be induced by DNA damaging agents [1]–[4]. DSBs must be repaired properly to maintain cell viability and to prevent genome instability. A number of inherited human diseases associated with genome instability and cancer are found to be caused by mutations in the DNA DSB repair pathways. For instance, Mre11, Nbs1, ATM and BRCA1, which play critical roles in DNA damage checkpoint and DSB repair are linked ataxia-telangiectasia-like disorder (ATLD), Nijmegen breakage syndrome (NBS), ataxia-telangiectasia and familial breast cancer, respectively [5]–[9]. However, the exact mechanisms of how these proteins regulate DSB repair in mammalian cells are not fully understood.

DSBs can be repaired by Ku-dependent non-homologous end joining (NHEJ) and homologous recombination (HR)-mediated DSB repair [10], [11]. While NHEJ is active in all phases of the cell cycle, HR is activated only when cells enter S-phase, which is mainly attributed to the essential function of cyclin-dependent kinases (CDKs) to promote end resection at DSB ends [12]. NHEJ can be error-prone, but HR is highly accurate in preserving all genetic information by using the identical sister chromatids as templates to repair DSBs.

The Mre11/Rad50/Nbs1 (MRN) complex binds to DSB ends and plays important roles in facilitating ATM activation and promoting end resection to initiate HR-mediated DSB repair [13]–[15]. CtIP (CtBP-interacting protein), which is associated with MRN and BRCA1, is also a critical player in the regulation of HR [16]–[20]. In mammalian cells, CtIP binds to MRN through Nbs1 [16], [18], [21]. Such interaction was also observed in fission yeast, where the FHA/BRCT domains of Nbs1 interact with the phospho-motifs at the CK2 sites on Ctp1, and this interaction is important for recruiting Ctp1 to DSB ends by Nbs1 [22], [23]. In budding yeast, the CtIP homologue Sae2 exhibits endonuclease activities to cleave the ssDNA (single-strand DNA)/dsDNA (double-strand DNA) junctions and the sites close to DNA hairpin loops [24]. Although the nuclease activity of CtIP itself was not identified, CtIP was found to promote the nuclease activity of MRN to process DSB ends in mammalian cells [16].

Various studies suggest that CtIP and its homologues are important for regulating end resection during the cell cycle [16], [17], [25]–[27]. CtIP is directly phosphorylated by CDKs, and phosphorylation of CtIP at the CDK site S327 induces its interaction with BRCA1, which is important for HR-mediated DSB repair [18], [20], [26]. Furthermore, phosphorylation of a conserved CDK site S267 on Sae2 is essential for HR in budding yeast [27], and its corresponding site T847 on CtIP is also required for end resection in mammalian cells [17], although the mechanisms underlying this regulation are not clear.

In this study, we identified a new cluster of CDK sites situated in the middle of CtIP and demonstrated that phosphorylation of these CDK sites is a prerequisite for ATM to phosphorylate CtIP upon DNA damage. We also showed that ATM-mediated phosphorylation of CtIP, mainly through the newly identified conserved site T859, is essential for promoting end resection and HR, suggesting a mechanism of how CDK-mediated phosphorylation activates damage-induced HR. We further showed that phosphorylation of these identified CDK sites on CtIP induces associations of CtIP with the FHA/BRCT domains of Nbs1. Interestingly, this CDK-dependent interaction of CtIP with the FHA/BRCT domains of Nbs1 facilitates ATM to phosphorylate CtIP, thereby coupling CDK-phosphorylation with ATM-mediated phosphorylation of CtIP to promote end resection and HR.

## Results

### CtIP is phosphorylated by ATM in response to DNA damage

**Figure 1:**
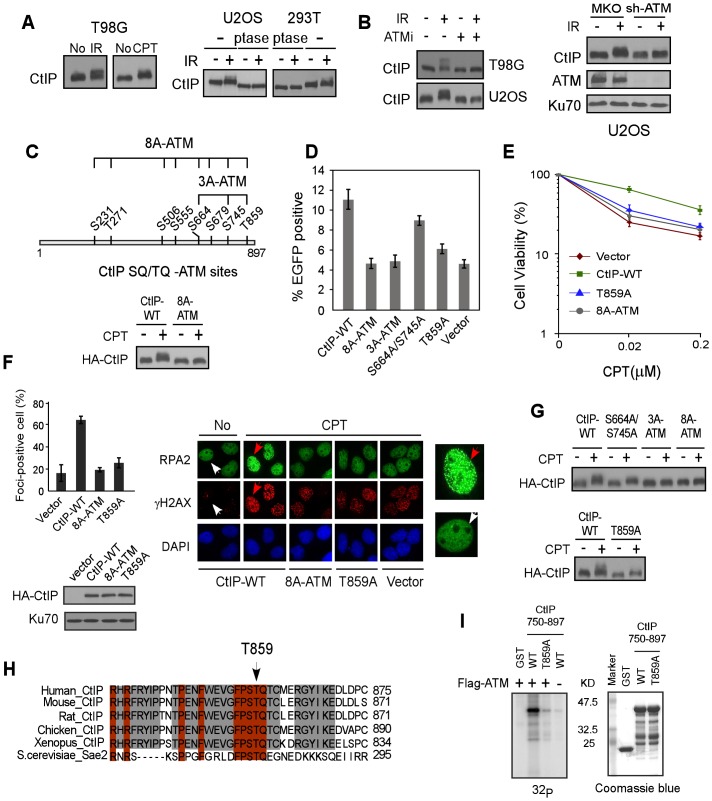
ATM is important for CtIP hyper-phosphorylation after DNA damage. A. Indicated cell lines were treated with IR (10 Gy) or CPT (2 µM, 2 h) or No and lysed. Western blotting of CtIP was performed using cell lysates incubated with lambda phosphatase (ptase) or without ptase (-). B. U2OS and/or T98G cells were treated with ATM inhibitor (ATMi) KU-55933 (20 µM, 1 h) or infected with retroviruses encoding shRNAs for ATM or vector control MKO, treated with IR (10 Gy) and lysed 1 h later. Western blotting was performed with indicated antibodies. C. Schematic drawing of eight putative ATM consensus sites (SQ/TQ motifs) on CtIP. U2OS cells stably expressing HA-CtIP wild-type (WT) or HA-CtIP 8A-ATM mutant (all SQ/TQ sites mutated to AQ) with endogenous CtIP silenced by shRNAs and siRNA, were treated with CPT (2 µM, 2 h) followed by anti-HA Western blotting. D–F. EGFP-based HR assay, clonogenic survival and RPA foci formation assays were performed with U2OS cells stably expressing indicated CtIP variants, with endogenous CtIP silenced. Data shown represents the mean of three independent experiments; error bars, s.d. In panel F, indicated cells were treated with DMSO (No) or CPT (1 µM, 2 h) and fixed for immunostaining. The red and white arrows indicate representative RPA2 and γH2AX foci-positive and foci-negative cells, respectively, with RPA2-immunostained cells enlarged in right-side panels. The percentage of RPA2 foci-positive cells among γH2AX-positive cells for each sample is shown. G. Western blot of U2OS cells expressing indicated CtIP variants with endogenous CtIP silenced and treated with CPT (2 µM, 2 h). H. The C-terminus protein sequence of human CtIP was aligned with CtIP homologues from other indicated species, with newly identified conserved T859 site shown. I. ATM *in vitro* kinase assay was performed with purified GST-tagged CtIP fragments (residues 750–897), containing either WT or the T859A mutation. Coomassie Blue staining indicates protein loading.

DNA damage induces CtIP phosphorylation, as revealed by phosphatase-sensitive mobility shift of CtIP on SDS-PAGE after ionization radiation (IR) or camptothecin (CPT) treatment (Figure 1A and data not shown). By using ATM inhibitor (ATMi) KU-55933 or expressing ATM shRNAs, we found that IR- and CPT-induced CtIP phosphorylation is dependent on ATM (Figure 1B and data not shown). *In vitro* kinase assays showed that purified CtIP can be phosphorylated by ATM (Figure S1A). Mutating all eight putative ATM kinase sites (SQ/TQ) on CtIP (CtIP-8A-ATM) completely abolished damage-induced CtIP phosphorylation shift (Figure 1C), further supporting that damage-induced phosphorylation is mediated by ATM.

### A newly identified conserved TQ site T859 on CtIP is phosphorylated by ATM and is important for end resection and HR

By using EGFP-based HR assays (Figure S1B), we demonstrated that the CtIP-8A-ATM mutant is significantly impaired in HR (Figure 1D). We further showed that the CtIP-S664A/S745A/T859A (CtIP-3A-ATM) mutant carrying mutations at three C-terminal SQ/TQ sites exhibits a strong defect in HR, but not the mutants CtIP-S231A/T271A and CtIP-S506A/S555A/S679A (Figure S1C and Figure 1D). While mutating the previously described S664 and S745 sites caused limited HR defect (Figure 1D, [28]), a single site mutation at T859 strongly reduced HR. Consistently, the CtIP-T859A mutant exhibited strong sensitivity to CPT, and reduced CPT-induced RPA foci formation (Figure 1E and 1F). These data suggest that ATM-mediated phosphorylation on CtIP mainly at T859 is important for promoting end resection and HR-mediated DSB repair.

Mutating S664 and S745 or T859 alone reduced, but mutating all three S664, S745 and T859 sites (CtIP-3A-ATM) almost completely abolished damage-induced phosphorylation as revealed by damage-induced phosphorylation shift on SDS-PAGE (Figure 1G), suggesting that the ATM sites S664, S745 and T859 are indeed phosphorylated *in vivo*. Alignment of CtIP from different species revealed that the newly identified TQ site T859 is conserved (Figure 1H). *In vitro* kinase assay showed that ATM phosphorylates the wild-type CtIP-750-897 fragment but not the fragment carrying T859A mutation, suggesting that ATM can directly phosphorylate this site (Figure 1I). Collectively, our studies suggest that ATM-mediated phosphorylation of CtIP is important for end resection during HR, and the conserved ATM site T859 identified in this study is critical for this function.

### CDK-mediated phosphorylation of CtIP is required for ATM to phosphorylate CtIP upon DNA damage

**Figure 2:**
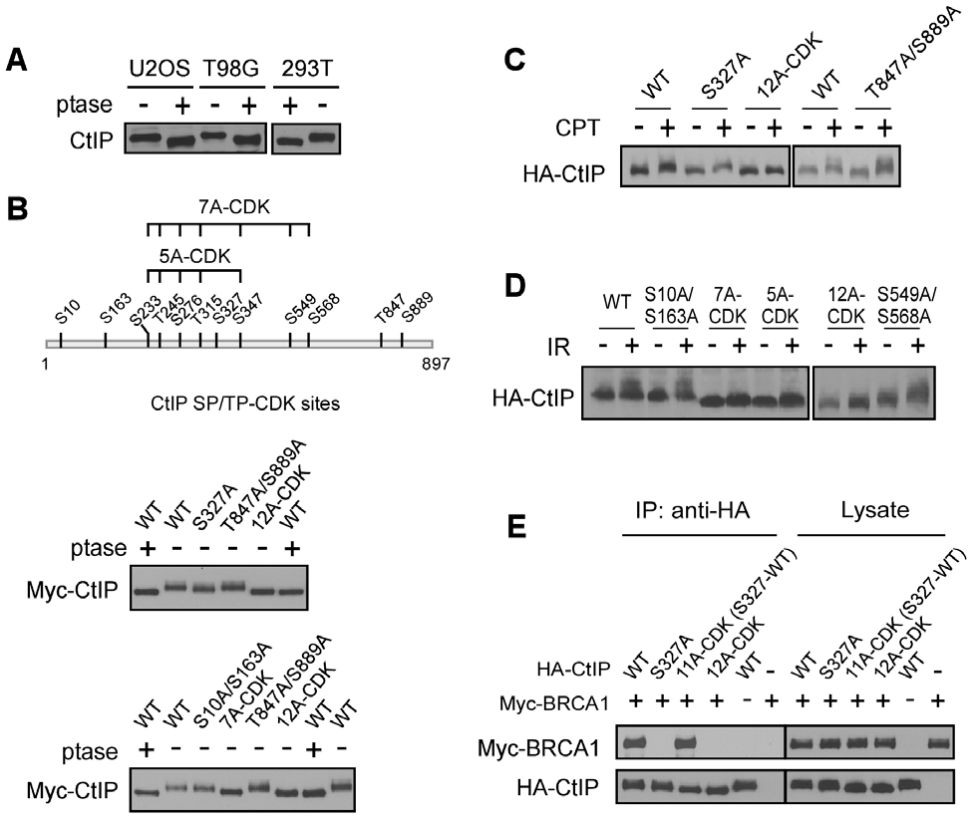
CtIP is phosphorylated by CDKs at multiple sites. A. Lysates from indicated cell lines were collected and treated with (+) or without (−) lambda phosphatase (ptase), followed by immunoblotting for endogenous CtIP. B. Schematic drawing of twelve putative CDK consensus sites (SP/TP motifs) on CtIP, with the middle cluster of sites 7A-CDK and 5A-CDK indicated. Myc-CtIP WT or indicated mutants were expressed in 293T cells, and cell lysates were treated with or without lambda phosphatase, followed by anti-Myc immunoblotting. C. and D. U2OS cells stably expressing HA-CtIP WT or indicated mutants with endogenous CtIP silenced, were treated with or without CPT (2 µM, 2 h) or IR (10 Gy, recovered for 1 h), followed by immunoblotting for anti-HA. E. Myc-BRCA1 and/or HA-CtIP WT or indicated mutants were expressed in 293T cells and co-immunoprecipitation was performed.

Treating cell lysates with phosphatase or mutating 12 putative CDK sites (SP/TP sites) increased CtIP mobility on SDS-PAGE to a similar level (Figure 2A and Figure 2B, middle panel), suggesting that CtIP is mainly phosphorylated by CDKs during the cell cycle. However, the CtIP mutants S327A or T847A/S889A only slightly reduced the phosphorylation-dependent shift (Figure 2B, middle and bottom panels), suggesting that CDK sites other than previously identified S327 and T847 [17], [20] are also phosphorylated *in vivo*. Indeed, mutating a middle cluster of seven CDK sites (CtIP-7A-CDK: S233A, T245A, S276A, T315A, S347A, S549A and S568A, excluding S327) almost completely abolished the CtIP shift (Figure 2B, bottom panel).

To confirm that the CDK sites on CtIP are indeed phosphorylated *in vivo*, we performed mass spectrometry analysis of endogenous CtIP purified from HeLa cells. Peptides containing phospho-Ser and phospho-Thr at multiple CDK putative SP/TP sites including S10, S163, S233, T245, S276, T315, S327, S347, S549, S568 and S889 were recovered, confirming that these putative CDK sites are indeed phosphorylated *in vivo* (Table S1). However, the previously identified CDK site T847 was not revealed in this analysis [17], which may be attributed to instability of the phosphorylation at this site.

Strikingly, upon DNA damage including CPT and IR, the CtIP-12A-CDK mutant failed to be further phosphorylated by ATM as revealed by mobility shift on SDS-PAGE (Figure 2C and 2D), suggesting that CDK-mediated CtIP phosphorylation is a prerequisite for CtIP to be phosphorylated after DNA damage. Further analysis showed that the CDK-site mutants S10A/S163A, S327A, S549A/S568A and T847A/S889A exhibited normal damage-induced CtIP phosphorylation, but CtIP-5A-CDK (S233A, T245A, S276A, T315A and S347A) and CtIP-7A-CDK mutants were strongly impaired in this damage-induced phosphorylation (Figure 2C and 2D). These studies suggest that phosphorylation of the 5 CDK sites situated in the middle of CtIP (5mCDK sites: 5 middle CDK sites S233, T245, S276, T315 and S347) is required for damage-induced phosphorylation of CtIP by ATM. By synchronizing T98G cells through serum starvation, we showed that IR-induced CtIP hyper-phosphorylation does not occur in G1 cells and only occurs when cells enter S-phase (Figure S2). These data further support that CDK activity is important for damage-induced CtIP phosphorylation by ATM. Furthermore, we showed that while S327 is critical for BRCA1 binding [20], mutating the other 11 CDK sites on CtIP [all CDK sites except S327: 11A-CDK(S327-WT)] does not influence BRCA1 association (Figure 2E), suggesting that the interaction of CtIP with BRCA1 is not required for regulating damage-induced CtIP phosphorylation.

### CDK-mediated CtIP phosphorylation is important for end resection and HR

**Figure 3:**
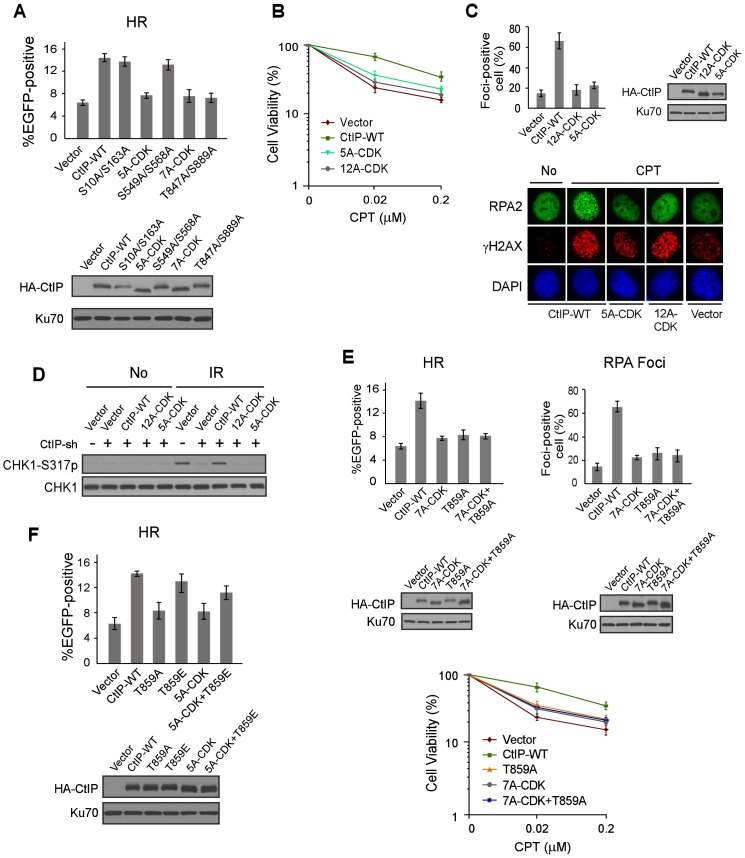
ATM-mediated phosphorylation of CtIP is important for end resection in a CDK-dependent manner. A. EGFP-HR assay was performed with U2OS cells stably expressing HA-CtIP WT or indicated mutants, with endogenous CtIP silenced. Data represent the mean ± s.d. of three independent experiments. Western blot shows expression of HA-CtIP variants. B. Clonogenic survival assay after treatment with indicated doses of CPT (1 h) was performed with U2OS cells stably expressing indicated CtIP variants with endogenous CtIP silenced. Data represent the mean ± s.d. of three independent experiments. C. RPA foci formation was examined in U2OS cells with indicated CtIP variants, before or after CPT treatment. The percentage of RPA foci-positive cells was determined as in Figure 1F. D. U2OS cells stably expressing indicated CtIP variants with endogenous CtIP silenced were treated with IR (10 Gy, recovered for 1 h), lysed, and immunoblotting was performed. E. EGFP-HR repair assay, clonogenic survival and RPA foci formation assays were performed in U2OS cells expressing HA-CtIP-WT or indicated mutants with endogenous CtIP silenced. F. EGFP-HR assay was performed with U2OS cells stably expressing HA-CtIP WT or indicated mutants, with endogenous CtIP silenced. Data represent the mean ± s.d. of three independent experiments. Western blot shows expression of HA-CtIP variants.

Consistent with that CDK-mediated phosphorylation of the middle cluster of SP/TP sites is required for ATM to phosphorylate CtIP upon DNA damage, the CDK mutants CtIP-5A-CDK and CtIP-7A-CDK were found to be defective in HR to a similar level as the CtIP-T847A/S889A mutant containing the previously mapped CDK site T847 [17], while the mutants CtIP-S10A/S163A and S549A/S568A did not exhibit such defects (Figure 3A). In addition, the CDK mutants CtIP-5A-CDK and CtIP-12A-CDK were sensitive to CPT (Figure 3B). As revealed by damage-induced RPA foci formation, end resection was defective in the CtIP-5A-CDK mutant to a level comparable to the CtIP-12A-CDK mutant with all CDK sites mutated (Figure 3C), and consequently IR-induced ATR activation as judged by Chk1 phosphorylation was also compromised (Figure 3D). These studies suggest that the 5mCDKs are important for end resection and HR-mediated DSB repair.

To show that CDK-mediated phosphorylation of CtIP is in the same pathway as ATM-mediated CtIP phosphorylation to regulate end resection and HR, we combined the mutations of CtIP-7A-CDK with the ATM site mutation T859A, and the resultant mutant CtIP-7A-CDK/T859A exhibited similar defects in HR, end resection and CPT sensitivity as the separate CDK and ATM mutants, CtIP-7A-CDK and CtIP-T859A (Figure 3E). These data support the conclusion that HR defects observed in the CtIP-7A-CDK mutant is due to its impaired function to promote ATM-mediated CtIP phosphorylation upon DNA damage. To determine whether CtIP phosphorylation by ATM can bypass the requirement of CDK-mediated phosphorylation of CtIP, we introduced the phospho-mimic mutation T859E to the CtIP-5A-CDK mutant. The HR defects observed in the CtIP-5A-CDK were largely suppressed in the CtIP-5A-CDK/T859E mutant (Figure 3F). The interaction of Nbs1 with CtIP phospho-mimic mutants CtIP-8E-ATM (mutating all 8 SQ/TQ sites to EQ) and CtIP-T859E remains at the similar levels as wild-type CtIP (Figure S4C). These data further support that CDK-mediated phosphorylation of CtIP is important for CtIP to be phosphorylated by ATM to mediate the DNA damage response.

### Nbs1 promotes ATM-mediated phosphorylation of CtIP through its FHA/BRCT domains in a CDK phosphorylation-dependent manner

**Figure 4:**
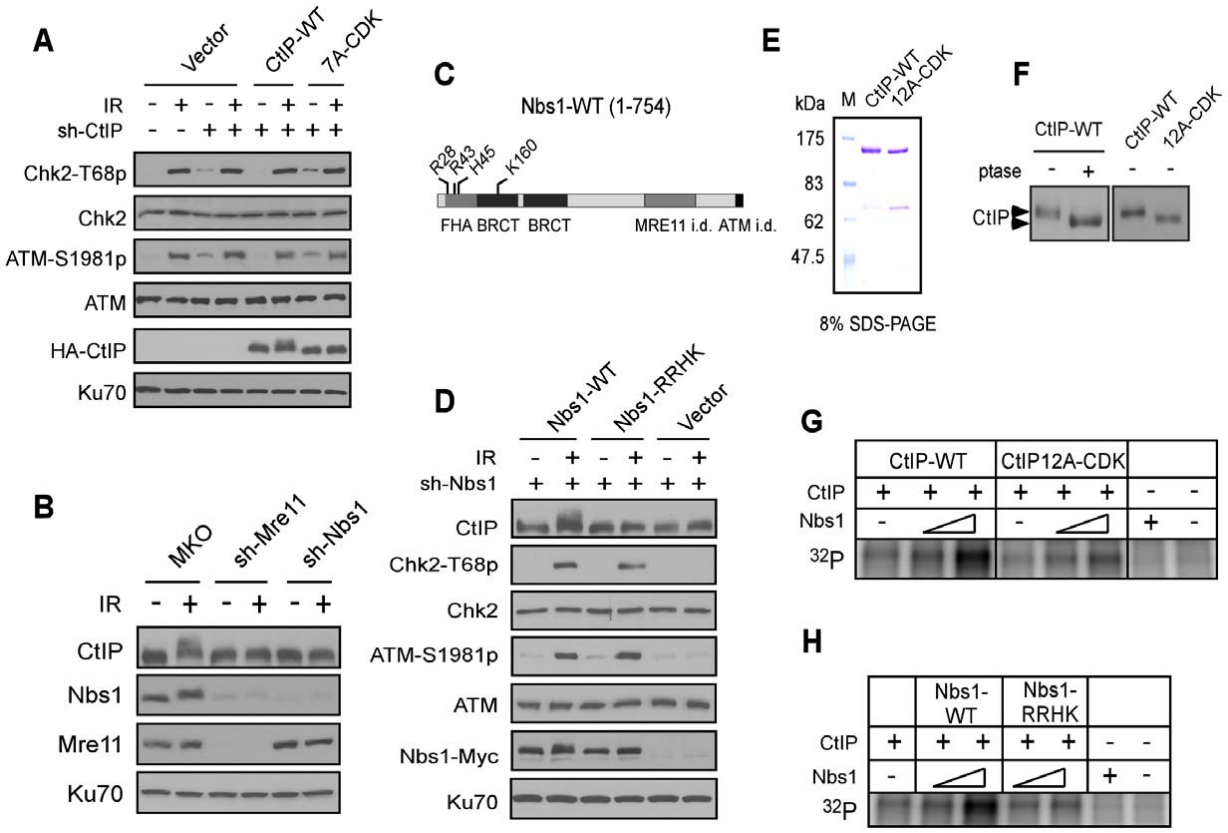
The FHA/BRCT domains of Nbs1 are important for ATM-mediated phosphorylation of CtIP. A. The CtIP-7A-CDK mutant does not show defects in ATM and checkpoint activation. U2OS cells stably expressing HA-CtIP WT or 7A-CDK mutant, with endogenous CtIP silenced, were treated with or without IR (10 Gy) and allowed to recover for 1 h. Cells were then collected and lysed for Western blot analysis with indicated antibodies. B. U2OS cells stably expressing control vector MKO, sh-Mre11 or sh-Nbs1 were treated with IR (10 Gy, recovered for 1 h), lysed, and immunoblotting was performed. C. Schematic drawing of human Nbs1 protein domains [FHA, BRCT and Mre11- and ATM interacting domains (i.d.)], with indicated phospho-binding sites on the FHA/BRCT domains [31], [32]. D. U2OS cells stably expressing C-terminus Myc-tagged Nbs1 alleles, Nbs1-WT and Nbs1-RRHK (R28A, R43A, H45A and K160M) or empty vector with endogenous Nbs1 silenced by shRNAs were treated with or without IR (10 Gy, recovered for 1 h), lysed, and immunoblotting was performed. E. Purified GST-CtIP WT and GST-CtIP-12A-CDK from Sf9 insect cells were analyzed by SDS-PAGE with Coomassie blue staining. F. CtIP expressed in insect cells is phosphorylated by CDKs. Purified GST-CtIP WT and 12A-CDK mutant protein from insect cells were treated without or with lambda phosphatase (ptase), and anti-CtIP Western blot analysis was performed. G and H. Baculovirus-expressed and purified CtIP-WT and CtIP-12A-CDK mutant proteins (100 ng) were used as substrates for *in vitro* ATM kinase assays with or without addition of purified Nbs1-WT or Nbs1-RRHK mutant proteins (100 ng and 500 ng). The radiolabeled CtIP was visualized following SDS-PAGE.

Our studies suggest that CDK-mediated phosphorylation of CtIP directly or indirectly modulates damage-induced phosphorylation of CtIP by ATM. Consistent with the previous findings that CtIP is dispensable for ATM activation [16], [29], no defects of ATM activation were detected in the CtIP-7A-CDK-mutant as revealed by damage-induced ATM auto-phosphorylation and Chk2 phosphorylation (Figure 4A). As with many other ATM substrates, damage-induced CtIP phophorylation depends on Nbs1 and Mre11 (Figure 4B), likely due to the role of MRN in promoting ATM activation [30]. Interestingly, however, when we used an Nbs1 FHA/BRCT mutant, Nbs1-RRHK (R28A/R43A/H45A/K160A) carrying mutations at the critical sites for binding to the phospho-motifs in the Nbs1 FHA/BRCT domains [31], [32], we found that damage-induced CtIP phosphorylation was impaired, but ATM auto-phosphorylation and ATM-mediated phosphorylation of Chk2 was normal (Figure 4D). This suggests that the FHA/BRCT domains of Nbs1 are not required for ATM activation *per se* but are specifically required for ATM to phosphorylate CtIP in response to DNA damage.

To further understand the role of Nbs1 to facilitate ATM-mediated phosphorylation of CtIP, we performed *in vitro* ATM kinase assays using purified GST-CtIP-His (Figure 4E). Human CtIP, expressed in insect cells but not in bacteria, was phosphorylated by CDKs as revealed by reduced mobility on SDS-PAGE upon phosphatase treatment or after mutating 12 putative CDK sites on CtIP (Figure 4F). We also performed mass spectrometry analysis and found that the same CDK sites are phosphorylated on CtIP when expressed in insect cells as in mammalian cells (Table S2). Although ATM can phosphorylate CtIP *in vitro* (Figure S1A), addition of Nbs1, purified from insect cells (Figure S3A), significantly promoted such ATM-mediated phosphorylation of CtIP when less amounts of CtIP and ATM were used (Figure 4G). Interestingly, this Nbs1-promoted CtIP phosphorylation by ATM was observed only when CtIP-WT, but not CtIP-12A-CDK was used. Furthermore, the Nbs1 FHA/BRCT mutant Nbs1-RRHK also failed to promote ATM-mediated phosphorylation of CtIP *in vitro* (Figure 4H). These studies suggest that Nbs1 promotes ATM phosphorylation of CtIP in a manner dependent on CDK-phosphorylation of CtIP and the FHA/BRCT domains of Nbs1.

### The FHA/BRCT domains of Nbs1 interact with CtIP through the middle cluster of CDK sites, which promotes ATM-mediated phosphorylation of CtIP

**Figure 5:**
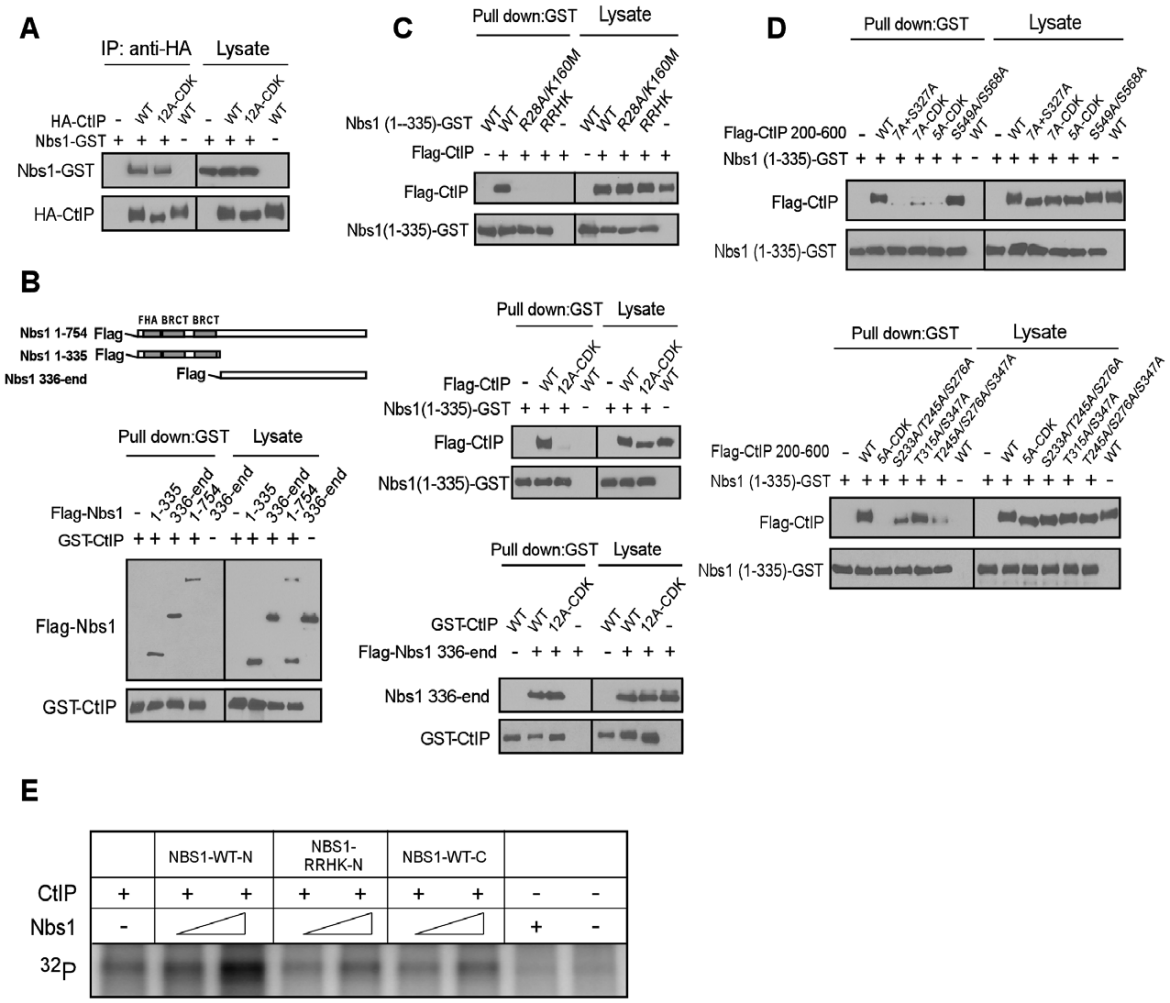
CtIP interacts with the FHA/BRCT domains of Nbs1 in a CDK phosphorylation-dependent manner. A to D. Characterizing the interaction of CtIP and Nbs1. Indicated Nbs1 and CtIP constructs were co-expressed in Sf21 insect cells by baculovirus infection, followed by anti-HA IP or GST pull-down assays and immunoblotting. *A*. Full-length CtIP-12A-CDK mutant associates with Nbs1 containing C-terminal GST-tag. *B*. Both the N-terminus and C-terminus of Nbs1 bind to CtIP. *C*. *Top*: The phospho-binding sites in the Nbs1 FHA/BRCT domains are important for mediating the interaction with CtIP. *Middle*: The CtIP-12A-CDK mutant fails to bind to the Nbs1 FHA/BRCT domains. *Bottom*: Both CtIP-WT and CtIP-12A-CDK mutant interact with the C-terminus of Nbs1. *D*. The CtIP 5mCDK sites are important for interaction with Nbs1 FHA/BRCT domains. *Top*: The CtIP 200–600 fragment containing the 5A-CDK mutations fails to bind to Nbs1 FHA/BRCT domains. *Bottom*: The 5mCDK sites redundantly mediate the interaction of CtIP with Nbs1 FHA-BRCT domains. E. Purified CtIP (100 ng) was used for *in vitro* ATM kinase assays in the presence or absence of Nbs1-WT-N (residues 1–335), Nbs1-RRHK-N mutant (residues 1–335) or Nbs1-WT-C (residues 336-end) (100 ng and 500 ng).

FHA and BRCT domains often mediate the binding of phospho-proteins through specific interactions of phosphorylated motifs [33]–[35]. To understand how Nbs1 facilitates CtIP phosphorylation by ATM through its FHA/BRCT domains in a CDK phosphorylation-dependent manner, we characterized the interactions of Nbs1 with CtIP. As shown in Figure 5A, full-length Nbs1 binds to CtIP-WT and CtIP-12A-CDK at similar levels. Further analysis revealed that both the N-terminus of Nbs1 [Nbs1-(1–335), containing the FHA/BRCT domains] and C-terminus of Nbs1 [Nbs1-(336-end)] bind to CtIP (Figure 5B), suggesting that CtIP and Nbs1 interact at more than one site.

Strikingly, the Nbs1-(1–335)-GST fragment containing the FHA/BRCT domains with GST tagged at the C-terminus, but not the corresponding FHA/BRCT mutants Nbs1-(1–335)-R28A/K160M-GST and Nbs1-(1–335)-RRHK-GST, bind CtIP efficiently (Figure 5C, top). Furthermore, Nbs1-(1–335)-GST binds to Flag-tagged CtIP-WT but not the CtIP-CDK-12A mutant (Figure 5C, middle), while the C-terminus Nbs1-(336-end) fragment binds to CtIP-WT and CtIP-12A-CDK at approximately equal levels (Figure 5C, bottom). By using purified proteins, we further demonstrated that the interaction of Nbs1-(1–335) with CtIP is direct, and such interaction can be disrupted by the RRHK mutations in the Nbs1 FHA domain and the CDK-site mutations (12A-CDK) in CtIP (Figure S3B).

We also found that Nbs1-(1–335), but not Nbs1-(1–335)-RRHK interacts with the middle region of CtIP [CtIP-(200–600)], and mutating all CDK sites (7A+S327A) in this CtIP-(200–600) fragment abolished the interactions (Figure S4A and Figure 5D top). On the other hand, the C-terminus Nbs1-(336-end) fragment binds to the C-terminus of CtIP [CtIP-(462-end)] (Figure S4B). These studies suggest that Nbs1 interacts with CtIP through two distinct means: one is through the FHA/BRCT domains of Nbs1 to bind to the middle cluster of CDK phospho-motifs on CtIP, and the other is a constitutive interaction through the C-termini of Nbs1 and CtIP.

To further analyze CDK-phosphorylation-mediated interaction of CtIP with the FHA/BRCT domains of Nbs1, we mutated different CDK sites in the CtIP-(200–600) fragment. While the CtIP- (200–600)-S549A/S568A mutant still binds to the Nbs1 FHA/BRCT domains [Nbs1-(1–335)-GST], the CtIP-(200–600) fragment containing the 7A-CDK or 5A-CDK mutation fails to bind (Figure 5D, top), suggesting that the 5mCDK sites are important for mediating the interactions with the Nbs1 FHA/BRCT domains. We then separated the mutations in the 5A-CDK mutant, and found that the CtIP-(200–600) fragments containing mutations of S233A/S245A/S276A, T315A/S347A or T245A/S276A/S347A all reduce, but do not abolish the binding to Nbs1-(1–335) (Figure 5D, bottom). It has been suggested that the FHA domain mediates phosphorylated threonine binding, while the BRCT domains often bind to phosphorylated serines [36], [37]. We thus examined the interaction of Nbs1-(1–335)-R28A-GST carrying mutations in the FHA domain with the CtIP-(200–600)-S233A/S276A/S347A mutant, and found that the interaction of the BRCT domains with CtIP is abolished by non-phosphorylation mutations at these three serine residues (Figure S5A, left). The interaction of Nbs1-(1–335)-K160M-GST carrying mutations in the second BRCT domain with CtIP-(200–600)-T245A/T315A was also reduced (Figure S5A, right). These data suggest that S233, S276 and S347 are important for binding to the BRCT domains, and T245 and T315 contributes to the binding to the Nbs1 FHA domain. Consistent with that both FHA and BRCT domains of Nbs1 are involved in the interactions with the CDK sites on CtIP, we also showed that introducing mutations to both FHA and BRCT domains of Nbs1 in the Nbs1-(1–335) fragment (Nbs1-RRHK and Nbs1-R28A/K160M) abolished its interactions with CtIP-(200–600), while mutating either FHA domain (Nbs1-R28A) or BRCT domain (Nbs1-K160M) only reduced the interaction (Figure S5B).

We demonstrated that full-length Nbs1 promotes ATM to phosphorylate CtIP in a manner dependent on the FHA/BRCT domains of Nbs1 and CDK-phosphorylation of CtIP (Figure 4G and 4H). To examine whether this activity depends on the Nbs1 and CtIP interaction, purified Nbs1-(1–335) or Nbs1-(336-end) was added into the *in vitro* ATM kinase assay (Figure S3A). Interestingly, only Nbs1-(1–335) but not Nbs1-(336-end) promoted ATM to phosphorylate CtIP, and Nbs1-(1–335) carrying the FHA/BRCT mutations did not show this Nbs1-dependent stimulation of ATM phosphorylation of CtIP (Figure 5E). These data suggest that the specific interactions of the FHA/BRCT domains of Nbs1 with 5mCDK sites on CtIP modulate CtIP phosphorylation by ATM.

### ATM-mediated phosphorylation of CtIP is important for promoting recruitment of BLM and Exo1 to DSBs to initiate HR

**Figure 6:**
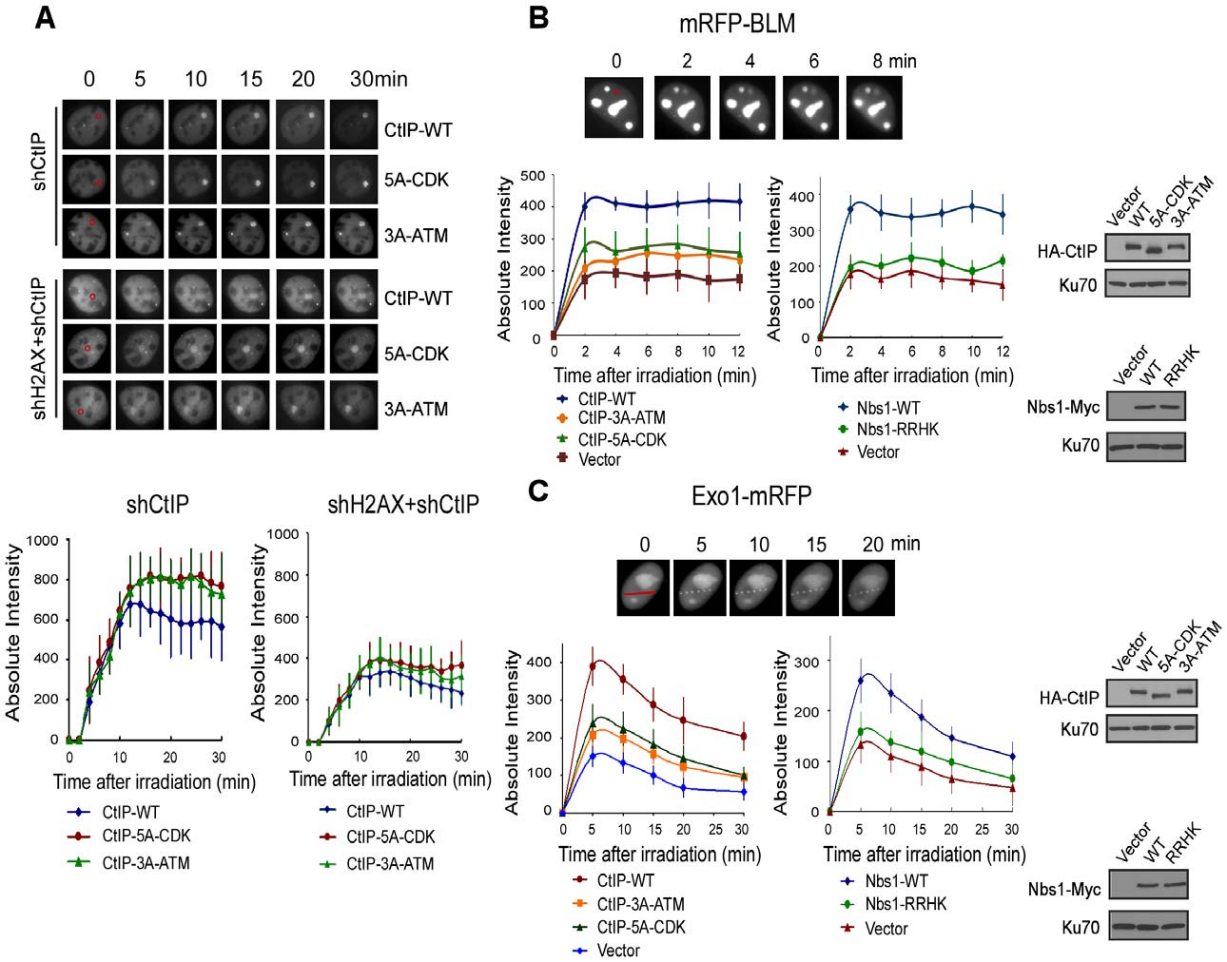
ATM-mediated phosphorylation of CtIP promotes the recruitment of BLM and Exo1 to laser-induced DSBs. A. EGFP-CtIP variants and mRFP-PCNA were co-expressed in U2OS cells with endogenous CtIP or both CtIP and H2AX silenced by shRNAs. Live-cell imaging of EGFP-CtIP in S-phase cells, marked by PCNA S-phase-associated replication foci [68], was performed following laser-induced microirradiation. Representative cells show the recruitment of EGFP-CtIP WT or indicated mutants to laser-microirradiated disk regions, which are indicated by red circles in the 0 min cell images. Absolute intensity of EGFP-CtIP fluorescence signals at damage sites was determined; error bars, s.d. B and C. Recruitment of mRFP-BLM (B) and Exo1-mRFP (C) to DSBs was monitored in U2OS cells expressing HA-CtIP or Nbs1-Myc variants, with endogenous CtIP or Nbs1 silenced. Representative cells show the recruitment of mRFP-BLM or Exo1-mRFP to microirradiated regions (a disk region for mRFP-BLM marked by a red circle and dots on a line for Exo1-mRFP marked by a red line in 0 min cell images). Absolute intensities of mRFP-BLM or Exo1-mRFP fluorescence signals were determined; error bars, s.d. Western blots show expression of indicated proteins, with Ku70 as a loading control.

In fission yeast, the interaction of Nbs1 FHA/BRCT domains with the CK2 sites on Ctp1 is required for Ctp1 to be recruited to DSBs [23]. In mammalian cells, CtIP recruitment to DSBs depends on Nbs1 and ATM [29]. To monitor whether CDK- and ATM-mediated phosphorylation regulates CtIP recruitment, we performed live-cell imaging to examine the recruitment of EGFP-tagged CtIP to DSBs upon laser microirradiation. With endogenous CtIP silenced by shRNAs, we showed that the initial recruitments of the CtIP-5A-CDK and CtIP-3A-ATM (S664A/S745A/T859A) mutants to chromosomal DSBs are comparable to CtIP-WT (Figure 6A). However at later time points, CtIP-WT started to disassociate from DSBs faster than the CtIP-5A-CDK and CtIP-3A-ATM mutants, likely due to compromised DSB repair in these mutants. We also monitored CtIP localization to DSB ends in the absence of γH2AX-dependent recruitment to DSB-flanking regions. As expected, inactivation of H2AX significantly reduced CtIP recruitment to laser-generated DSB-containing regions, but γH2AX-independent recruitment of CtIP-5A-CDK and CtIP-3A-ATM mutants to DSB ends was also similar to that of CtIP-WT at the initial stages after laser treatment (Figure 6A). Thus, CDK-phosphorylation of CtIP is not necessarily required for CtIP recruitment to DSBs in mammalian cells.

BLM and Exo1 play important roles in end resection to promote HR, and the recruitments of BLM and Exo1 to DSBs are dependent on CtIP (Figure 6B, 6C: compare CtIP-WT and vector, [38]–[41]). By monitoring mRFP-tagged BLM and Exo1 localization to chromosomal DSBs in live cells, we found that the recruitment of BLM and Exo1 to DSBs was significantly reduced in the CtIP-5A-CDK and CtIP-3A-ATM mutant cell lines (Figure 6B left and 6C left). Furthermore, BLM and Exo1 recruitments were also compromised when the FHA/BRCT domains of Nbs1 are mutated (Figure 6B right and 6C right). These observations suggest that ATM-mediated phosphorylation of CtIP, which requires the interactions of the FHA/BRCT domains of Nbs1 with the 5mCDK sites, is important for promoting the recruitment of BLM and Exo1 to DSBs. Impaired recruitment of BLM and Exo1 to DSBs is thus an important factor causing the defects of the CtIP CDK- and ATM-phosphorylation mutants in end resection and HR.

### The interactions of the Nbs1 FHA/BRCT domains with CtIP and with MDC1 are involved in different mechanisms to repair DSBs

**Figure 7:**
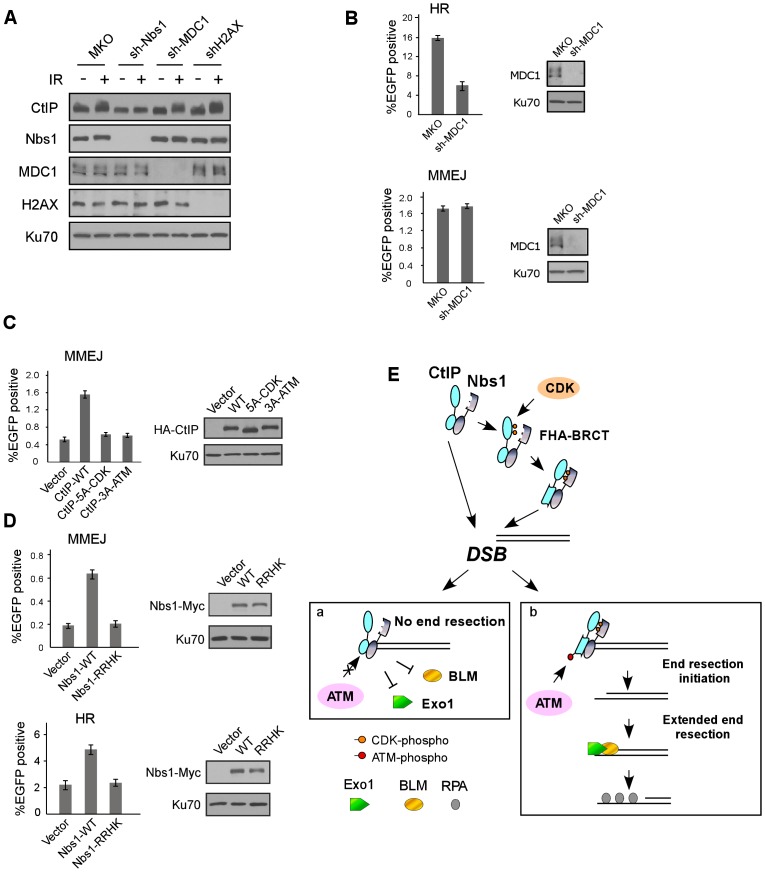
The interactions of Nbs1 FHA/BRCT domains with CtIP or with MDC1 involve different mechanisms to regulate DSB repair. A. U2OS cells stably expressing control MKO or indicated shRNAs were treated with or without IR (10 Gy, recovered for 1 h), lysed, and immunoblotting was performed. B. EGFP-HR and EGFP-MMEJ assays were performed with U2OS cells stably expressing MKO or sh-MDC1. Western blotting was performed to show silencing of MDC1, with Ku70 as a loading control. C. EGFP-MMEJ assay was performed in U2OS cells expressing indicated CtIP variants, with endogenous CtIP silenced. Western blotting shows expression of HA-CtIP variants with Ku70 as a loading control. D. EGFP-MMEJ and EGFP-HR assays were performed in U2OS cells expressing Nbs1-WT-Myc or Nbs1-RRHK-Myc mutant with endogenous Nbs1 silenced. Western blotting shows expression of Nbs1-Myc variants with Ku70 as a loading control. E. A model to describe a role for CDK- and ATM-mediated phosphorylation of CtIP in the regulation of end resection and DSB repair (see Discussion).

It was described that the Nbs1 FHA/BRCT domains bind to MDC1 through multiple phosphorylated CK2 sites on MDC1 in mammalian cells [42]–[45]. The binding of Nbs1 to multiple CtIP CDK sites shows similar characteristics, where the 5mCDK sites redundantly contribute to the binding. We thus compared the role of Nbs1/MDC1 and Nbs1/CtIP complexes in DSB repair. First, the interaction of the Nbs1 FHA/BRCT domains with phosphorylated CK2 sites on MDC1 is important for Nbs1 to be recruited to DSB-flanking site in a γH2AX-dependent manner [42]–[46]. However, we found that ATM-dependent phosphorylation of CtIP depends on the Nbs1 FHA/BRCT domains, but not on MDC1 and H2AX (Figure 4D and Figure 7A), suggesting that the interactions of the FHA/BRCT domains of Nbs1 with CtIP at CDK sites likely occur directly at DSB ends independently of γH2AX and MDC1 to promote ATM-mediated phosphorylation of CtIP. Therefore, the Nbs1/CtIP and Nbs1/MDC1 complexes are likely formed at different chromosomal locations with respect to the DNA lesions. In addition, overexpression of MDC1 does not influence ATM-mediated phosphorylation of CtIP (Figure S6A), and thus does not affect the interaction of Nbs1 with CtIP, which further supports that the interactions of Nbs1 with CtIP and with MDC1 are independent events. Second, we found that although both Nbs1/MDC1 and Nbs1/CtIP complexes are needed for HR, they play different roles in microhomology-mediated end joining (MMEJ), which is a major pathway of Ku-independent alternative NHEJ (alt-NHEJ, [47], [48]). To monitor MMEJ, we used an EGFP-based repair substrate with 9-bp duplication around the I-Sce1 cleavage site (Figure S6B and [49]). While HR was significantly reduced when MDC1 was inactivated by shRNAs, MMEJ was not affected (Figure 7B). However, when we expressed the CDK mutant CtIP-5A-CDK or CtIP-3A-ATM with endogenous CtIP silenced by shRNAs, both HR and MMEJ were reduced (Figure 7C, Figure 1D and Figure 3A). Consistently, both MMEJ and HR were reduced in the Nbs1 FHA/BRCT domain mutant Nbs1-RRHK with endogenous Nbs1 inactivated by shRNAs (Figure 7D). These data suggest that the associations of the FHA/BRCT domains of Nbs1 with CtIP and with MDC1 are engaged in different mechanisms to regulate DSB repair. While the Nbs1/MDC1 complex is important only for HR, the Nbs1/CtIP complex is needed for both MMEJ and HR.

## Discussion

Substantial evidence suggests that CDKs play a critical role in promoting end resection to activate HR, and CtIP is an important target of CDKs in this regulation [14], [17], [26], [50]. In addition to previously identified CDK sites S327 and T847 [17], [20], we identified novel CDK phosphorylation sites on CtIP and found that the FHA/BRCT domains of Nbs1 interact with these sites after CDK phosphorylation, which in turn promotes ATM-mediated phosphorylation of CtIP upon DNA damage to activate end resection and HR. Our further analyses showed that although the CDK sites S327, T847 and the newly identified 5mCDK sites are all involved in regulating CtIP function in end resection, the underlying mechanisms are different. While the CtIP-5A-CDK mutant is severely impaired in ATM-mediated phosphorylation of CtIP, mutating S327 or T847 does not show such defect. On the other hand, loss of CDK phosphorylation at S327 almost completely abolishes CtIP interaction with BRCA1, but the CtIP-5A-CDK and T847A mutants do not show defects in BRCA1 association. Therefore, CDK-mediated phosphorylation of CtIP regulates end resection through multiple levels of regulation.

We identified a novel ATM phosphorylation site T859 and showed that this site is conserved and located in the conserved RHR motif at the C-terminal Sae2-like domain [16], [25]. Along with two previously mapped ATM phosphorylation sites S664 and S745 [28], we found that T859 plays an important role in activating end resection and HR, suggesting that CtIP is a critical target for ATM to regulate end resection and HR. We further showed that phospho-mimic mutation at T859 (T859E) can bypass the requirement of CDK-mediated phosphorylation of CtIP for HR (Figure 3F), suggesting an important role of CDK-mediated phosphorylation of CtIP to promote CtIP phosphorylation by ATM at T859 to mediate DNA damage response. Damage-induced phosphorylation of Sae2 and Ctp1 was also observed in yeast and the phosphorylation is dependent on Tel1 and Mec1/Rad3 [51], [52]. Since ATM-mediated phosphorylation of CtIP does not require functional MDC1 and H2AX (Figure 7A), CtIP is likely phosphorylated by ATM at DSB ends independent of γH2AX-mediated recruitment of repair proteins to DSB-flanking chromatin.

It is intriguing that CDK-mediated phosphorylation promotes ATM to phosphorylate CtIP upon DNA damage. In this aspect, we found that in addition to a constitutive binding of Nbs1 with CtIP through the C-terminus of both proteins, phosphorylation of 5mCDK sites on CtIP induces a new interaction between these CDK sites with the FHA/BRCT domains of Nbs1. By using *in vitro* ATM kinase assays, we demonstrated that Nbs1 promotes ATM to phosphorylate CtIP in a manner dependent on its FHA/BRCT domains as well as the CDK sites on CtIP, suggesting that the interactions of the FHA/BRCT domains of Nbs1 with phosphorylated 5mCDK sites regulate ATM phosphorylation of CtIP. Since mutating the middle cluster of CDK sites on CtIP and the FHA/BRCT domains on Nbs1 does not affect ATM activation *per se* and phosphorylation of other ATM substrates such as Chk2 (Figure 4A and 4D), this CDK-dependent interaction of CtIP with Nbs1 is specifically required for facilitating ATM to phosphorylate CtIP. Based on these studies, we propose that upon DNA damage, CtIP is recruited to DSBs through constitutive interactions with MRN at the C-terminus of Nbs1 (Figure 7E). However, only those CtIP species that have been phosphorylated at the 5mCDK sites form additional associations with the FHA/BRCT domains of Nbs1, which may trigger conformational changes of CtIP and convert CtIP to a more favorable substrate for ATM by inducing exposure of the ATM phosphorylation sites such as S664, S745 and T859 at the C-terminus of CtIP. After being phosphorylated by ATM, CtIP is activated and functions to promote end resection and HR. Since CDK-mediated phosphorylation occurs only when cells enter S-phase, the requirement of CDK-dependent association of CtIP with Nbs1 to promote CtIP phosphorylation by ATM couples cell cycle regulation with DNA damage responses.

Based on the studies in yeast, it was proposed that DNA end resection is carried out via two steps: the initial end resection by the Mre11 complex and Sae2, and the extended end resection by Sgs1/Exo1 and Dna2 [53], [54]. In mammalian cells, it was described that BLM (human homologue of Sgs1) and Exo1 are important for promoting extended end resection to activate HR [39]–[41]. We observed that ATM-mediated phosphorylation of CtIP as well as CDK phosphorylation at 5mCDK sites on CtIP is required for the recruitments of BLM and Exo1 to DSBs. This study suggests that promoting BLM and Exo1 recruitment to DSBs is one important mechanism underlying ATM-mediated phosphorylation of CtIP to regulate end resection and HR. One possibility is that phosphorylation of CtIP by ATM promotes the initial end resection, which is required for BLM and Exo1 to be loaded onto DSBs to further promote end resection and activate HR (Figure 7E). In this aspect, we observed that ATM-mediated phosphorylation of CtIP is needed for both HR and MMEJ. In our designed MMEJ substrate, only limited end resection is needed to allow 9-bp microhomology to anneal for the end joining process, which implies that CtIP phosphorylation by ATM may be important for the initial limited end resection step. The exact mechanism of ATM activation of CtIP to promote initial end resection is still not clear. It is noteworthy that T859 is located in the conserved Sae2-like domain and ATM-mediated phosphorylation of T859 may modulate conformational change of this domain to activate CtIP function. Alternatively, ATM-mediated phosphorylation of CtIP may regulate the loading of BLM/Exo1 to DSBs through modulating protein-protein interactions. For instance, CtIP binds to Exo1 [38], and ATM-mediated phosphorylation of CtIP may modulate this interaction. It is also possible that ATM-mediated phosphorylation of CtIP induces CtIP to interact with other proteins, which in turn promote the recruitment of BLM and Exo1 to DSBs.

In mammalian cells, the FHA/BRCT domains of Nbs1 interact with phosphorylated CK2 sites on MDC1, which is important for Nbs1 recruitment to DSB-flanking regions [42]–[45]. In this study, we showed that the FHA/BRCT domains of human Nbs1 also bind to CtIP through the 5mCDK sites. Similar to MDC1 binding, the 5mCDK sites on CtIP redundantly mediate its interaction with the Nbs1 FHA/BRCT domains. Although sharing similar features, the bindings of the FHA/BRCT domains of Nbs1 with CtIP and with MDC1 appear to be independent and contribute to different modes of regulating DSB repair. While the Nbs1/MDC1 complex is recruited to the chromatin around DSBs by γH2AX, the Nbs1/CtIP complex likely binds directly to DSB ends and promotes ATM-mediated phosphorylation of CtIP in a manner independent of H2AX and MDC1. Furthermore, the CDK-dependent CtIP and Nbs1 interaction as well as ATM-mediated phosphorylation of CtIP are important for both HR and MMEJ, but MDC1 is only required for HR and not for MMEJ. One possibility for this difference is that the Nbs1/CtIP complex participates in both initial end resection required for MMEJ and subsequent extended end resection for HR, but the Nbs1/MDC1 complex only participates in the step to promote extended end resection.

Our studies identify new CDK sites on CtIP, and reveal a novel mechanism which couples CDK- and ATM-mediated phosphorylation of CtIP to promote end resection and HR. The involvement of multiple CDK phosphorylation events (S327, 5mCDK sites and T847) as well as ATM-mediated phosphorylation to regulate CtIP function in DSB repair supports the notion that CtIP is a key regulator for cell cycle-dependent selection of DSB repair pathways. The regulated interaction of CtIP and Nbs1 to modulate ATM-mediated phosphorylation of CtIP also provides new information as to how these proteins function coordinately with each other to promote appropriate DSB repair pathways during the cell cycle to avoid genome instability.

## Materials and Methods

### Cell culture and antibodies

Human U2OS, T98G, HeLa and 293T cells were cultured at 37°C in Dulbecco's modified eagle's medium with 10% fetal bovine serum (FBS) in the presence of antibiotics and 5% CO_2_. Insect cell line Sf21 was cultured in Grace's insect medium (Gibco) supplemented with 10% fetal bovine serum; Sf9 insect cell line was cultured in Sf-900II SFM medium (Gibco). T98G cells were synchronized via serum starvation by culturing cells in DMEM containing 0.1% FBS for 48 hours, followed by release into complete medium containing 10% FBS [55], [56]. Cell cycle profile analysis of synchronized T98G cells was performed as described [55], [56].

Monoclonal anti-CtIP antibody was generously provided by R. Bear [Columbia University, [57]. CtIP polyclonal antibodies were described previously [49]. The polyclonal antibodies against Mre11 (D27, 1∶500 dilution) and monoclonal anti-Nbs1 antibody (EE15, 1∶500 dilution) were described previously [18]. Other antibodies used include anti-Flag-M2 (Sigma, 1∶1000 dilution), anti-Myc-9E10 (Novus Biologicals, 1∶1000 dilution), anti-HA-11 (Covance, 1∶1000 dilution), anti-Ku70 (Santa Cruz Biotechnology, 1∶3000 dilution), anti-RPA2 (Oncogene, 1∶200 dilution), anti-H2AX-S319-p, anti-Chk1-S317-p (Cell Signaling Technology, 1∶200 and 1∶500 dilution), anti-Chk1 (R&D Systems, 1∶800 dilution), anti-Chk2-T68 (GeneTex, 1∶600 dilution), anti-Chk2 (Santa Cruz Biotechnology, 1∶800 dilution), anti-ATM (GeneTex, 1∶500 dilution), anti-ATM-S1981-p (GeneTex, 1∶500 dilution), anti-MDC1 (Sigma, 1∶500 dilution), anti-His (Invitrogen, 1∶500 dilution), anti-GAPDH (RDI, 1∶500 dilution).

### Plasmids, mutagenesis, and shRNA/RNAi

CtIP cDNA [20] was subcloned into pUC19 at BamHI/SalI and used to generate CtIP variants by site-directed mutagenesis (Stratagene). CtIP wild-type and indicated mutants were then subcloned into mammalian expression vectors pcDNA3 or pBabepuro containing HA, Myc or Flag epitopes. EGFP-tagged CtIP or indicated mutants were generated using EGFP-C1 expression vector (Clontech). GST-fused CtIP fragments were generated using pGEX4T-1 (GE Healthcare). Recombinant baculoviruses expressing GST-, HA- or Flag-tagged CtIP or Nbs1 were generated by using Bac-to-Bac Baculovirus expression systems (Invitrogen). Nbs1-RRHK mutant was made by site-directed mutagenesis (Stratagene), and Nbs1 truncation mutants were generated by PCR amplification of indicated fragments. Nbs1 wild-type and indicated mutants were subcloned into pBabepuro or pFastBAC-HTb (Invitrogen) vectors containing in-frame C-terminal Myc-, 10×His-, 6×His- or GST-tags. EGFP-Exo1 was a gift from Dr. Kum Kum Khanna [58] and used to make Exo1-mRFP-N1. mRFP-BLM was a gift from Dr. Marek Rusin [59]. HA-MDC1 was a gift from Dr. Jiri Lukas [42].

Silencing of endogenous CtIP by shRNAs and siRNAs was performed as described [49]. shRNA- and siRNA- resistant CtIP wild-type and indicated mutants were constructed by mutating four nucleotides at the shRNA/siRNA targeting sequences by site-directed mutagenesis (Stratagene). All other shRNAs were generated in pMKO vector using the following RNAi target sequences as designed by Dharmacon: for shH2AX GGGACGAAGCACUUGGUAA; for shMDC1 GUCUCCCAGAAGACAGUGAUU; for shNbs1 GAAGAAACGUGAACUCAAGUU; for shKu70 GAAGGAGGUUGCAGCAUUGUG and for shATM GGGCAUUACGGGUGUUGAA.

### Immunoprecipitation, *in vitro* binding assay, immunostaining, and clonogenic survival assay

Whole cell lysis, co-immunoprecipitation and Western blotting were performed as described [55]. Cells were lysed in NETN [150 mM NaCl, 1 mM EDTA, 20 mM Tris-HCl (pH 8.0), 0.5% NP-40] containing protease inhibitors. Immunoprecipitation was conducted by incubating primary antibodies with cell lysates at 4°C for 4 h, followed by the addition of protein A-agarose for one additional hour. For *in vitro* binding assay, GST-CtIP, Flag-Nbs1, Nbs1-GST, HA-CtIP or Flag-CtIP was expressed alone or in combination in insect cells, and binding assay was performed using NETN buffer.

For immunostaining, cells were cultured on sterile coverslips and treated with CPT as indicated. Cells were fixed with 4% paraformaldehyde, followed by immunostaining as described [18], [49]. After blocking with 5% goat serum in phosphate-buffered saline (PBS), fixed cells were incubated with indicated primary antibodies at 4°C overnight followed by incubation for 1 h at room temperature with either fluorescein isothiocyanate-conjugated anti-mouse and/or rhodamine-conjugated anti-rabbit secondary antibodies (Jackson ImmunoResearch Laboratories), followed by DAPI staining for nuclei. Fluorescence microscopy was performed using a Nikon Eclipse E800 upright fluorescence microscope (60×/1.4 oil Plan-APO objective, with FITC, Rhodamine and DAPI fluorochromes). Images were captured with a digital camera (AG Heinze, Co., Model RT-SE6) and analyzed using SPOT Advanced imaging software (Diagnostic Instruments, Inc).

For clonogenic survival assay, indicated cell lines were treated with camptothecin for 1 h, and allowed to culture in complete media for 10–14 days. Colonies were stained with a solution containing 0.5% crystal violet and 20% ethanol then counted, with the percentage of cell viability shown. Error bars are s.d. of three independent experiments [16].

### Homologous recombination (HR) and microhomology-mediated end-joining (MMEJ) assay

HR and MMEJ assays for DSB repair were described previously [49]. U2OS cells carrying EGFP-HR (Figure S1B) or EGFP-MMEJ (Figure S6B), with or without expressing indicated CtIP or Nbs1 variants, and/or indicated shRNAs, were transfected with I-SceI-IRES-dsRedNLS and plated in non-selective medium. After 72 h, cells were trypsinized and collected for fluorescence-activated cell sorting (FACS) analysis of EGFP-positive events, using a BD Accuri C6 flow cytometer and accompanying analysis software (Becton-Dickinson, MA, USA).

### Laser-induced micro-irradiation and live-cell imaging

U2OS cells expressing indicated EGFP- or mRFP-tagged proteins, with or without expressing indicated shRNAs, were cultured in sterile glass-bottom dishes (MatTek) in DMEM medium containing 10% FBS. Dishes were directly placed onto the microscope stage for image acquisition at room temperature and processed within a 1 h maximum time period for the experimental duration of any given dish. DSBs were generated in live-cell nuclei by laser-induced microirradiation using a picosecond short-pulsed green laser (a diode-pumped second harmonic 532 nm Nd∶YAG laser microbeam with 76 MHz repetition rate, 12 ps pulse duration). The average laser power used for DNA cutting was 16 milliwatts (post-objective), and the total energy delivered per focused laser spot was 480 mJ. Live-cell, time-lapse images were taken by the robotic laser microscopy system (RoboLase II) built on a Zeiss Axiovert 200 M inverted fluorescence microscope (63×,1.4 oil Plan-Apo objective, using FITC and Rhodamine fluorochromes) and digital camera (Hamamatsu ORCA-EA) [49], [60]. Images were captured using RoboLase II system software, and raw images (16-bit) were imported into ImageJ software (NIH of USA) for processing. Fluorescence intensities of the micro-irradiated area were determined by measuring the mean absolute intensity of the microirradiated areas, with the mean cellular background intensity subtracted [49], [61]. Each data point shown is the average of 10 independent measurements.

### Protein purification and *in vitro* kinase assay

GST-fused CtIP C-terminal region (residues 750–897) was expressed in BL21 and affinity-purified with glutathione-Sepharose 4B (GE Healthcare). CtIP wild-type and indicated mutants were expressed in Sf9 insect cells and purified by Ni-NTA and GST tandem affinity purification. C-terminal His-tagged Nbs1 wild-type and indicated mutant were expressed in Sf9 insect cells and purified by Ni-NTA column followed by MonoQ column. ATM *in vitro* kinase assays were previously described [49]. Briefly, FLAG-tagged ATM was transiently transfected into 293T cells and immunoprecipitated using anti-FLAG M2-agarose (Sigma). Immunoprecipitates were extensively washed and then incubated with purified proteins in the presence of 10 µCi γ-^32^P-ATP in ATM kinase buffer [25 mM HEPES (pH 7.4), 50 mM NaCl, 10 mM MnCl_2_, 10 mM MgCl_2_, 1 mM DTT, 5 µM ATP]. The kinase reaction was conducted at 30°C for 30 min. Phosphorylated proteins were separated by SDS-PAGE and visualized using a PhophorImage scanner (GE, Typhoon Trio). To test the effects of Nbs1 WT and mutants on CtIP phosphorylation by ATM *in vitro*, indicated amounts of CtIP and Nbs1 proteins were pre-incubated for 15 min on ice and then combined with Flag-ATM immunoprecipitates and γ-^32^P-ATP to perform the ATM kinase reaction.

### Mass spectrometry

Mass spectrometry was described before [62]. Five liters of HeLa cell suspension was lysed with NETN containing 1 µg/ml pepstatin A and aprotinin, and endogenous CtIP was affinity-purified for analysis using mammalian cells. For analysis using insect cells, recombinant human CtIP was expressed in SF9 insect cells, and purified by Ni-NTA and GST tandem affinity purification. Protein samples were precipitated by mixing cold sample solution (1 vol) with 1/3 vol of 100% (w/v) trichloroacetic acid (TCA) (6.1 N) to a final TCA concentration (conc.) of 25%, incubated on ice for 3 h, pelleted by cold centrifugation with acetone washes, and dried.

Proteins were dissolved in 60 µL of 100 mM Tris-HCl, pH 8.5 containing 8 M urea. The protein was reduced by adding 500 mM tris (2-carboxyethyl) phosphine to a final conc. of 5 mM, followed by carboxyamidomethylation of cysteines with 500 mM iodoacetamide (final conc. 10 mM). Protein was prepared for analysis of phosphorylation as described [62]. The protein sample was equally split for separate digestions by Subtilisin (Promega), Elastase, and Trypsin. The resulting peptides from the three digests were dissolved in 20% acetonitrile and 2% formic acid, combined, and subjected to TiO2 enrichment and LC-MS/MS analysis.

For TiO2 enrichment of phosphopeptide, a TiO2 column was made by pressure-slurry packing TiO2 (5-µ partisphere, Whatman, Clifton, NJ) into fused-silica capillary (250 µm i.d.). The column was washed with buffer A [water/acetonitrile/formic acid (95∶5∶0.1, v/v/v)] and buffer B [water/acetonitrile/formic acid (20∶80∶0.1, v/v/v)], then eluted using 250 mM ammonium bicarbonate onto an analytical column, which was then inserted into an Agilent 1200 quaternary HPLC pump for mass spectrometry analysis.

Data-dependent tandem mass spectrometry (MS/MS) analysis was performed with a LTQ-Orbitrap mass spectrometer (ThermoFisher). Full MS and tandem mass spectra were extracted and searched against an EBI-IPI human protein database (database released on June 28, 2007). A decoy database containing reversed sequences [63] was used to estimate peptide probabilities and identify false positives. Tandem mass spectra were matched to sequences using the ProLuCID algorithm [64].

ProLuCID searches were performed on an Intel Xeon 80-processor cluster (search tolerance set to 3 Da). The Cysteine mass was modified by +57.02146 Da to account for carboxyamidomethylation, and Serine, Threonine and Tyrosine were modified by +79.9663 Da for phosphorylation. Since no enzymatic cleavage conditions were imposed, the search included all candidates within the mass tolerance window, regardless of tryptic status [65]. Validation of peptide/spectrum matches (PSM) was assessed in DTASelect [66] using two SEQUEST [67] defined parameters, the cross-correlation score (XCorr), and normalized difference in cross-correlation scores (DeltaCN). Search results were grouped by charge state (+1, +2, +3, greater than +3) and tryptic status (full, half, and non) into 12 distinct sub-groups, and distribution of XCorr, DeltaCN, and DeltaMass values for direct and decoy database PSMs were obtained and separated by discriminant analysis. Peptide match probabilities were calculated based on a non-parametric fit of direct and decoy score distributions (minimum threshold of 90%). After filtering for false positives based on the percentage of reverse decoy PSMs, we estimate that both the protein and peptide false discovery rates were reduced to 0.0%–0.5%.

## Supporting Information

Figure S1 A. CtIP is phosphorylated by ATM. Purified CtIP protein (1 µg) was incubated with [γ-^32^P] ATP in the presence or absence of ATM kinase (immunoprecipitated from 293T by anti-Flag M2 beads). The radiolabeled CtIP was visualized following SDS-PAGE. Coomassie blue staining shows input of purified CtIP. B. Schematic representation of the EGFP-based HR repair assay substrate, EGFP-HR, as previously described [49]. A full-length EGFP cassette was disrupted by insertion of an I-SceI cleavage site containing two in-frame stop codons, followed by insertion of an inactive, truncated EGFP donor fragment (iEGFP) downstream of hygromycin resistance marker. Upon I-SceI induction of DSBs, HR-mediated repair using the iEGFP template generates a functional EGFP cassette. C. The CtIP-3A-ATM mutant (S664A/S745A/T859A) exhibits defect in HR repair. EGFP-HR assay was carried out in U2OS cells stably expressing indicated CtIP variants, with endogenous CtIP silenced by shRNAs and siRNA. Data shown represents the mean of three independent experiments; error bars, s.d. Western blot shows expression of HA-CtIP variants, with Ku70 used as a loading control. D. Western blot shows expression of HA-CtIP WT and indicated mutants used for the EGFP-HR assay shown in Figure 1D, with Ku70 used as a loading control.
(TIF)

Figure S2 T98G cells were synchronized in G0 by serum starvation, followed by release into complete media for 10 hr or 20 hr to obtain G1- or S-phase population cells, respectively. T98G cells, asynchronous (Asyn) or synchronized in G1 (10 hr) or S (20 hr), were treated with or without IR (10 Gy) at indicated time points after releasing from G0. Western blot analysis was performed using anti-CtIP antibody. The cell cycle profiles were determined by fluorescence activated cell sorted (FACS) analysis of propidium iodide stained cells.
(TIF)

Figure S3 A. *Left*: Schematic representation of the FHA and BRCT domains on Nbs1 and the Nbs1 mutants generated, including N-terminus (1–335) and C-terminus (336-end) truncation mutants, and/or point mutations in the FHA and BRCT domains. Interaction domain, i.d. *Right*: Purified Nbs1 WT and indicated mutant proteins were analyzed by SDS-PAGE gel with Coomassie blue staining. B. Purified Nbs1(1–335) or Nbs1(1–335)-RRHK (Figure S3A) were incubated with purified CtIP-WT or CtIP-12A-CDK coupled to Glutathione agarose beads. Western blot analysis was performed using anti-His and anti-GST antibodies.
(TIF)

Figure S4 A. The FHA/BRCT domains of Nbs1 (1–335) interact with the middle region of CtIP (200–600) in a CDK-phosphorylation dependent manner. *Top*: Schematic representation of full-length CtIP with twelve putative CDK consensus sites (SP/TP) indicated, and Flag-tagged N-terminal, middle and C-terminal CtIP fragments (amino acids 1–200, 200–600, and 600–897, respectively). *Bottom*: Sf21 insect cells were co-infected with baculoviruses expressing Nbs1-1–335-GST and indicated Flag-CtIP fragments with or without indicated CDK-site mutations. GST pull-down experiments were carried out, and immunoblotting was performed using anti-Flag M2 antibody. B. The C-terminus of Nbs1 (336-end) interacts with the C-terminus of CtIP (462-end). *Top*: Schematic representation of CtIP full-length and GST-tagged CtIP fragments (N-terminal fragment: amino acids 1–461; and C-terminal fragment: amino acids 462–897). *Bottom*: Sf21 insect cells were co-infected with baculoviruses expressing Flag-Nbs1 336-end, and GST-CtIP 1–461 or GST-CtIP 462-end fragments. GST pull-down experiments were carried out, and immunoblotting was performed using anti-Flag M2 antibody. C. CtIP phospho-mimic mutants 8E-ATM (all SQ/TQ sites mutated to EQ) and T859E were generated and co-expressed with Flag-tagged Nbs1 WT (full length) or 336-end in Sf21 insect cells by baculovirus infection, followed by GST pull-down or anti-M2 IP experiments and immunoblotting. *Top*, Both CtIP-WT and 8E-ATM mutant interact with full-length Nbs1. *Bottom*, T859E mutation does not affect the interaction between CtIP and Nbs1 C-terminus.
(TIF)

Figure S5 A. Indicated Nbs1 and CtIP variants were co-expressed in Sf21 insect cells by baculovirus infection, followed by GST pull-down experiments and immunoblotting. *Left*, Three serine site mutations, S233, S276, and S347, abolished the interaction between CtIP 200–600 with Nbs1 FHA domain mutant (Nbs1 1–335 R28A). *Right*, Two threonine site mutations, T245 and T315, reduced the interactions between CtIP 200–600 and Nbs1 BRCT domain mutant (Nbs1 1–335 K160M). B. Phospho-binding sites in the Nbs1 FHA/BRCT domains are important for its interaction with CtIP-(200–600) fragment. Sf21 insect cells were co-infected with baculoviruses co-expressing Nbs1-1–335-GST WT or indicated mutants with Flag-CtIP 200–600 fragment. GST pull-down experiments were carried out, and immunoblotting was performed using anti-Flag M2 antibody.
(TIF)

Figure S6 A. Overexpression of MDC1 does not affect CtIP hyper-phosphorylation by ATM. U2OS cells expressing vector control or HA-tagged MDC1 were treated with or without IR (10 Gy, recovered for 1 h), lysed, and immunoblotting was performed with indicated antibodies. B. Schematic drawing of the EGFP-MMEJ repair assay substrate, as previously described [49]. A full-length EGFP cassette was inactivated by inserting a 27-bp oligonucleotide containing an I-SceI cleavage site flanked on both sides by 9-bp microhomology sequence. Upon I-SceI induced generation of DSBs, limited end resection reveals the 9-bp microhomology region needed for annealing and repair of the DSB to generate a functional EGFP cassette. C. EGFP-MMEJ assays were performed with U2OS cells stably expressing control MKO, sh-Ku70 or both sh-Ku70 and sh-CtIP. Western blotting was performed to show silencing of KU70 and CtIP, with GAPDH as a loading control. D. EGFP-MMEJ assays were performed in U2OS cells stably expressing CtIP-WT or 3A-ATM mutant, with endogenous CtIP or both CtIP and Ku70 silenced by shRNAs. Relative repair frequencies were calculated by normalizing the percentage of induced repair to control (CtIP-WT with shCtIP), which is set to 1. Data shown represents the mean of three independent experiments; error bars, s.d. Western blot shows expression of HA-CtIP variants, with GAPDH as a loading control.
(TIF)

Table S1 Phosphorylated peptides of endogenous CtIP (from HeLa cells) were identified by mass spectrometry. All recovered peptide sequences are listed, with phosphorylated residues labeled with (p).
(PDF)

Table S2 Summary of phosphorylated peptides recovered by mass spectrometry analysis performed using HeLa cells expressing endogenous CtIP and SF9 insect cells expressing recombinant human CtIP (hCtIP). A plus (+) sign indicates that peptides were recovered from indicated, putative SP/TP sites, and a minus (-) sign indicates that peptides were not recovered from indicated site(s).
(PDF)
