# Supplementary figures and images for: The Interaction of CtIP and Nbs1 Connects CDK and ATM to Regulate HR–Mediated Double-Strand Break Repair
Source: PLoS Genet. 2013 Feb 28;9(2):e1003277. doi: 10.1371/journal.pgen.1003277 (PMC3585124; doi:10.1371/journal.pgen.1003277)

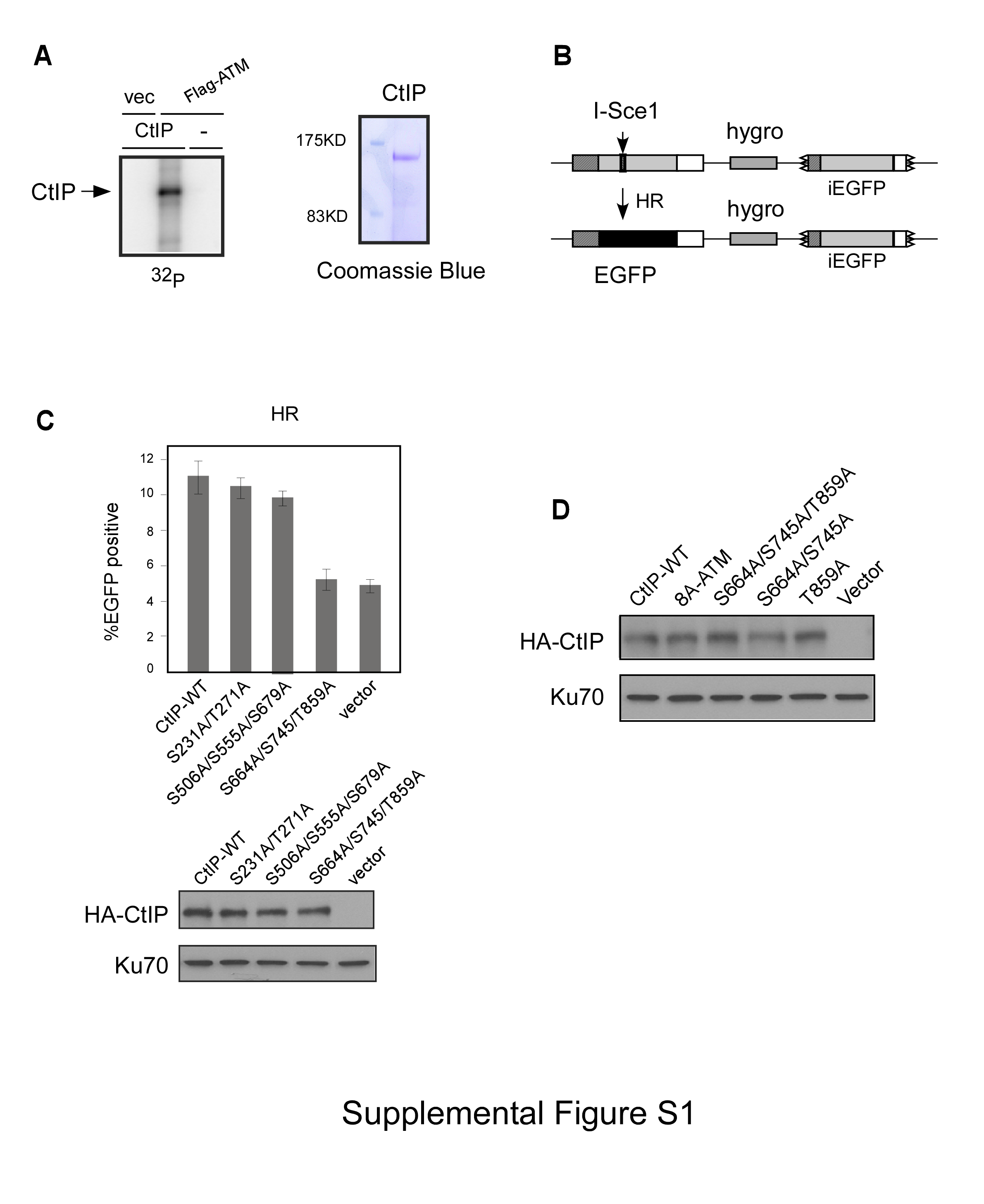

Supplement: Figure S1 — A. CtIP is phosphorylated by ATM. Purified CtIP protein (1 µg) was incubated with [γ-32P] ATP in the presence or absence of ATM kinase (immunoprecipitated from 293T by anti-Flag M2 beads). The radiolabeled CtIP was visualized following SDS-PAGE. Coomassie blue staining shows input of purified CtIP. B. Schematic representation of the EGFP-based HR repair assay substrate, EGFP-HR, as previously described [49]. A full-length EGFP cassette was disrupted by insertion of an I-SceI cleavage site containing two in-frame stop codons, followed by insertion of an inactive, truncated EGFP donor fragment (iEGFP) downstream of hygromycin resistance marker. Upon I-SceI induction of DSBs, HR-mediated repair using the iEGFP template generates a functional EGFP cassette. C. The CtIP-3A-ATM mutant (S664A/S745A/T859A) exhibits defect in HR repair. EGFP-HR assay was carried out in U2OS cells stably expressing indicated CtIP variants, with endogenous CtIP silenced by shRNAs and siRNA. Data shown represents the mean of three independent experiments; error bars, s.d. Western blot shows expression of HA-CtIP variants, with Ku70 used as a loading control. D. Western blot shows expression of HA-CtIP WT and indicated mutants used for the EGFP-HR assay shown in Figure 1D, with Ku70 used as a loading control. (TIF) [file pgen.1003277.s001.tif]

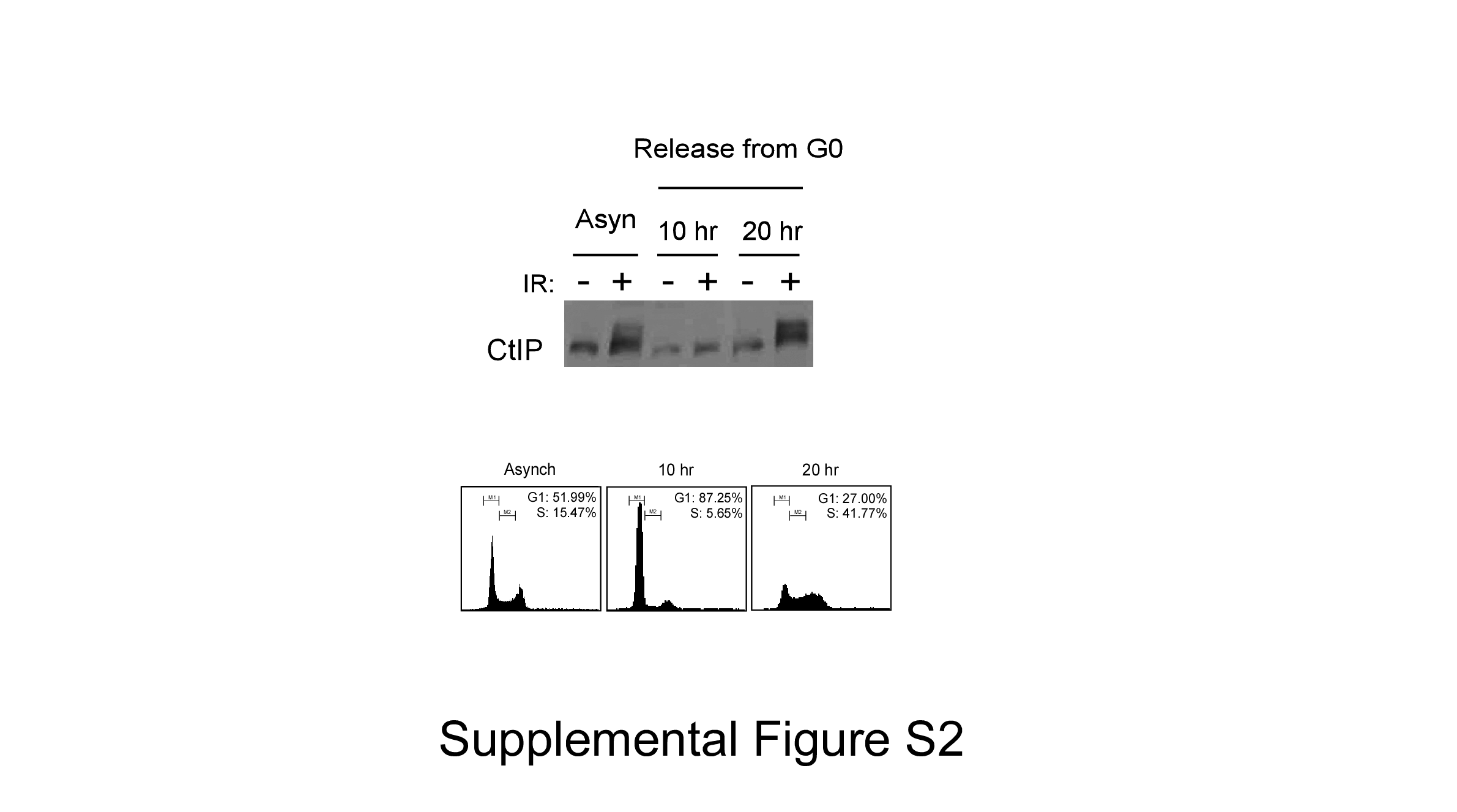

Supplement: Figure S2 — T98G cells were synchronized in G0 by serum starvation, followed by release into complete media for 10 hr or 20 hr to obtain G1- or S-phase population cells, respectively. T98G cells, asynchronous (Asyn) or synchronized in G1 (10 hr) or S (20 hr), were treated with or without IR (10 Gy) at indicated time points after releasing from G0. Western blot analysis was performed using anti-CtIP antibody. The cell cycle profiles were determined by fluorescence activated cell sorted (FACS) analysis of propidium iodide stained cells. (TIF) [file pgen.1003277.s002.tif]

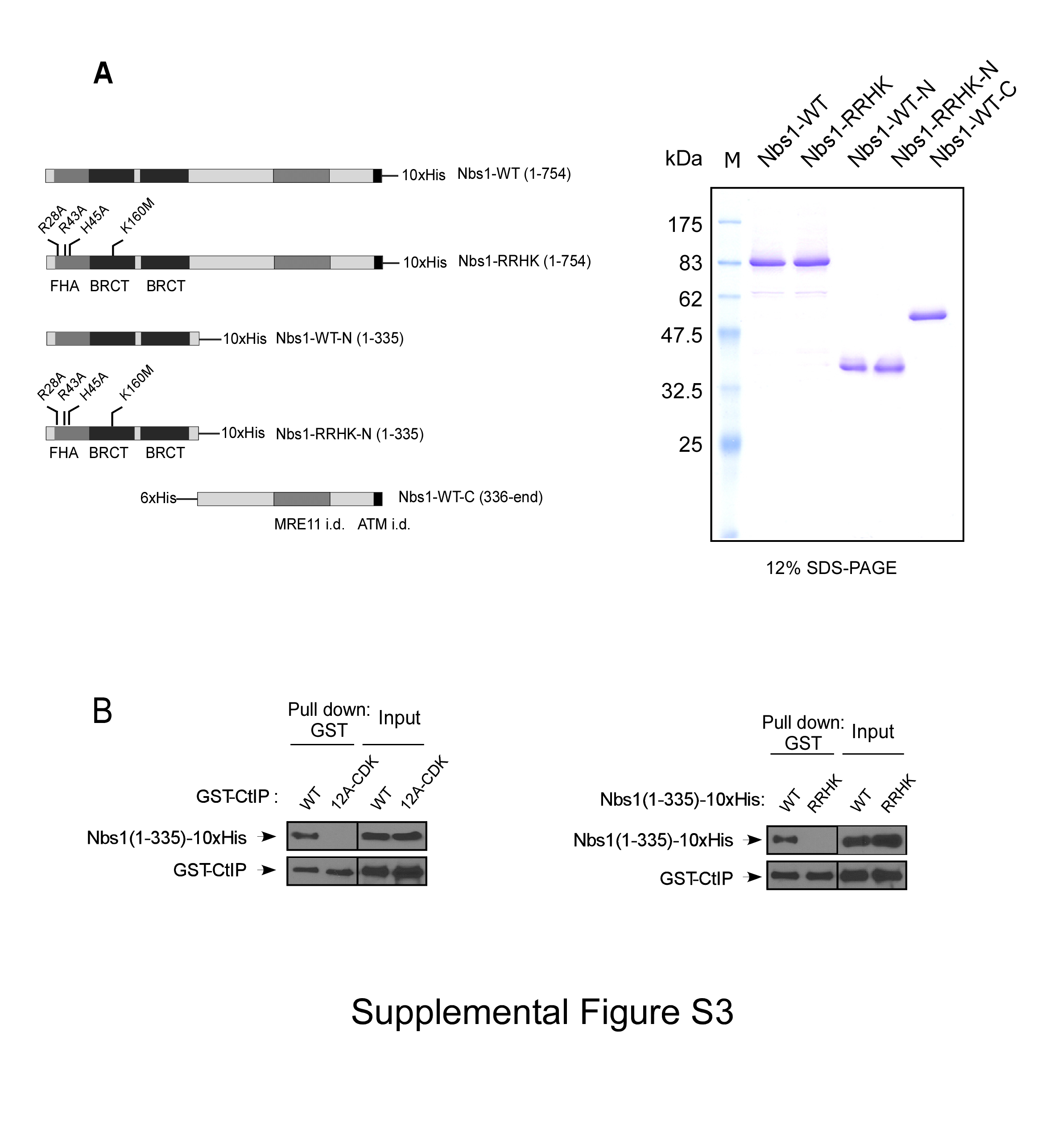

Supplement: Figure S3 — A. Left: Schematic representation of the FHA and BRCT domains on Nbs1 and the Nbs1 mutants generated, including N-terminus (1–335) and C-terminus (336-end) truncation mutants, and/or point mutations in the FHA and BRCT domains. Interaction domain, i.d. Right: Purified Nbs1 WT and indicated mutant proteins were analyzed by SDS-PAGE gel with Coomassie blue staining. B. Purified Nbs1(1–335) or Nbs1(1–335)-RRHK (Figure S3A) were incubated with purified CtIP-WT or CtIP-12A-CDK coupled to Glutathione agarose beads. Western blot analysis was performed using anti-His and anti-GST antibodies. (TIF) [file pgen.1003277.s003.tif]

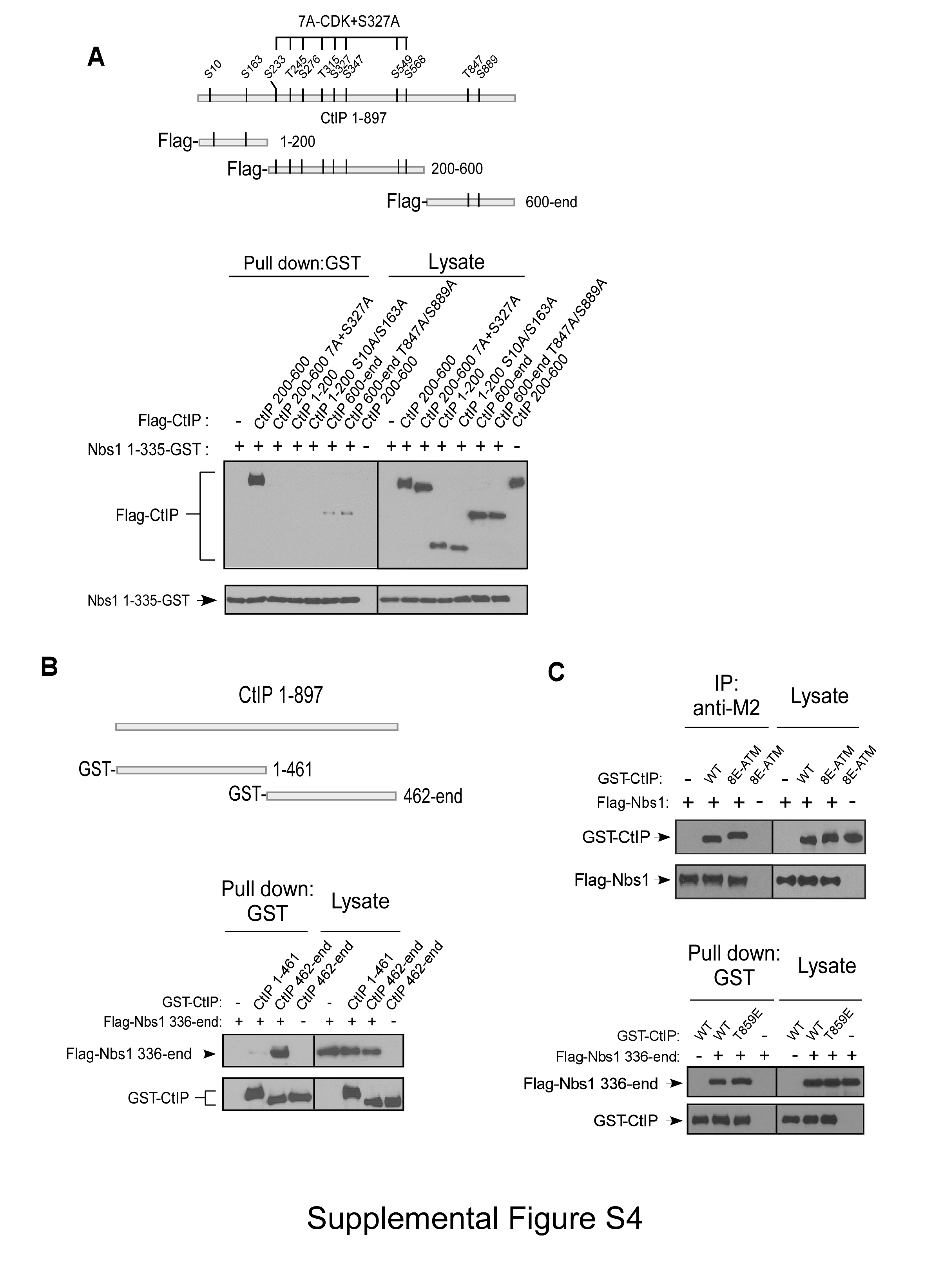

Supplement: Figure S4 — A. The FHA/BRCT domains of Nbs1 (1–335) interact with the middle region of CtIP (200–600) in a CDK-phosphorylation dependent manner. Top: Schematic representation of full-length CtIP with twelve putative CDK consensus sites (SP/TP) indicated, and Flag-tagged N-terminal, middle and C-terminal CtIP fragments (amino acids 1–200, 200–600, and 600–897, respectively). Bottom: Sf21 insect cells were co-infected with baculoviruses expressing Nbs1-1–335-GST and indicated Flag-CtIP fragments with or without indicated CDK-site mutations. GST pull-down experiments were carried out, and immunoblotting was performed using anti-Flag M2 antibody. B. The C-terminus of Nbs1 (336-end) interacts with the C-terminus of CtIP (462-end). Top: Schematic representation of CtIP full-length and GST-tagged CtIP fragments (N-terminal fragment: amino acids 1–461; and C-terminal fragment: amino acids 462–897). Bottom: Sf21 insect cells were co-infected with baculoviruses expressing Flag-Nbs1 336-end, and GST-CtIP 1–461 or GST-CtIP 462-end fragments. GST pull-down experiments were carried out, and immunoblotting was performed using anti-Flag M2 antibody. C. CtIP phospho-mimic mutants 8E-ATM (all SQ/TQ sites mutated to EQ) and T859E were generated and co-expressed with Flag-tagged Nbs1 WT (full length) or 336-end in Sf21 insect cells by baculovirus infection, followed by GST pull-down or anti-M2 IP experiments and immunoblotting. Top, Both CtIP-WT and 8E-ATM mutant interact with full-length Nbs1. Bottom, T859E mutation does not affect the interaction between CtIP and Nbs1 C-terminus. (TIF) [file pgen.1003277.s004.tif]

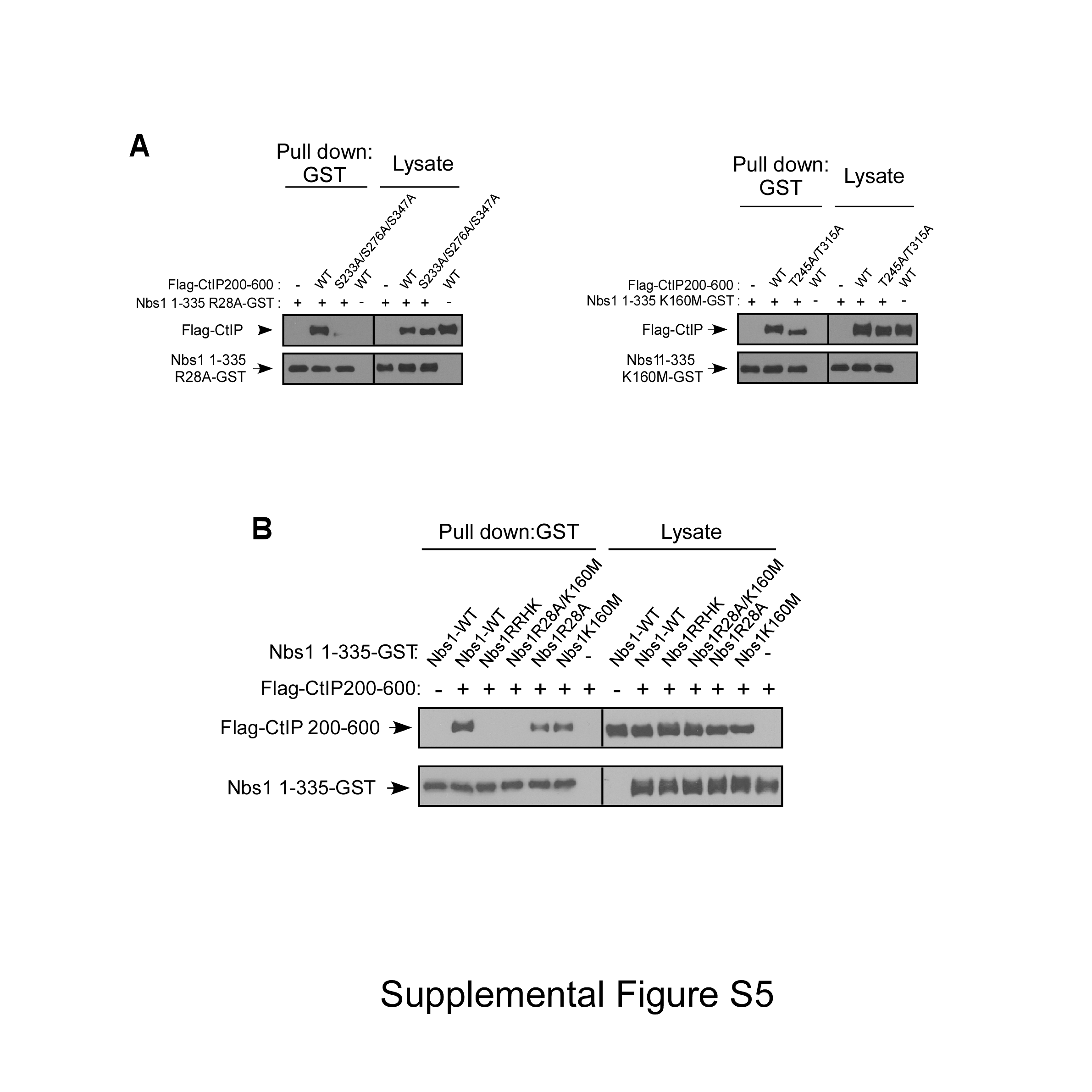

Supplement: Figure S5 — A. Indicated Nbs1 and CtIP variants were co-expressed in Sf21 insect cells by baculovirus infection, followed by GST pull-down experiments and immunoblotting. Left, Three serine site mutations, S233, S276, and S347, abolished the interaction between CtIP 200–600 with Nbs1 FHA domain mutant (Nbs1 1–335 R28A). Right, Two threonine site mutations, T245 and T315, reduced the interactions between CtIP 200–600 and Nbs1 BRCT domain mutant (Nbs1 1–335 K160M). B. Phospho-binding sites in the Nbs1 FHA/BRCT domains are important for its interaction with CtIP-(200–600) fragment. Sf21 insect cells were co-infected with baculoviruses co-expressing Nbs1-1–335-GST WT or indicated mutants with Flag-CtIP 200–600 fragment. GST pull-down experiments were carried out, and immunoblotting was performed using anti-Flag M2 antibody. (TIF) [file pgen.1003277.s005.tif]

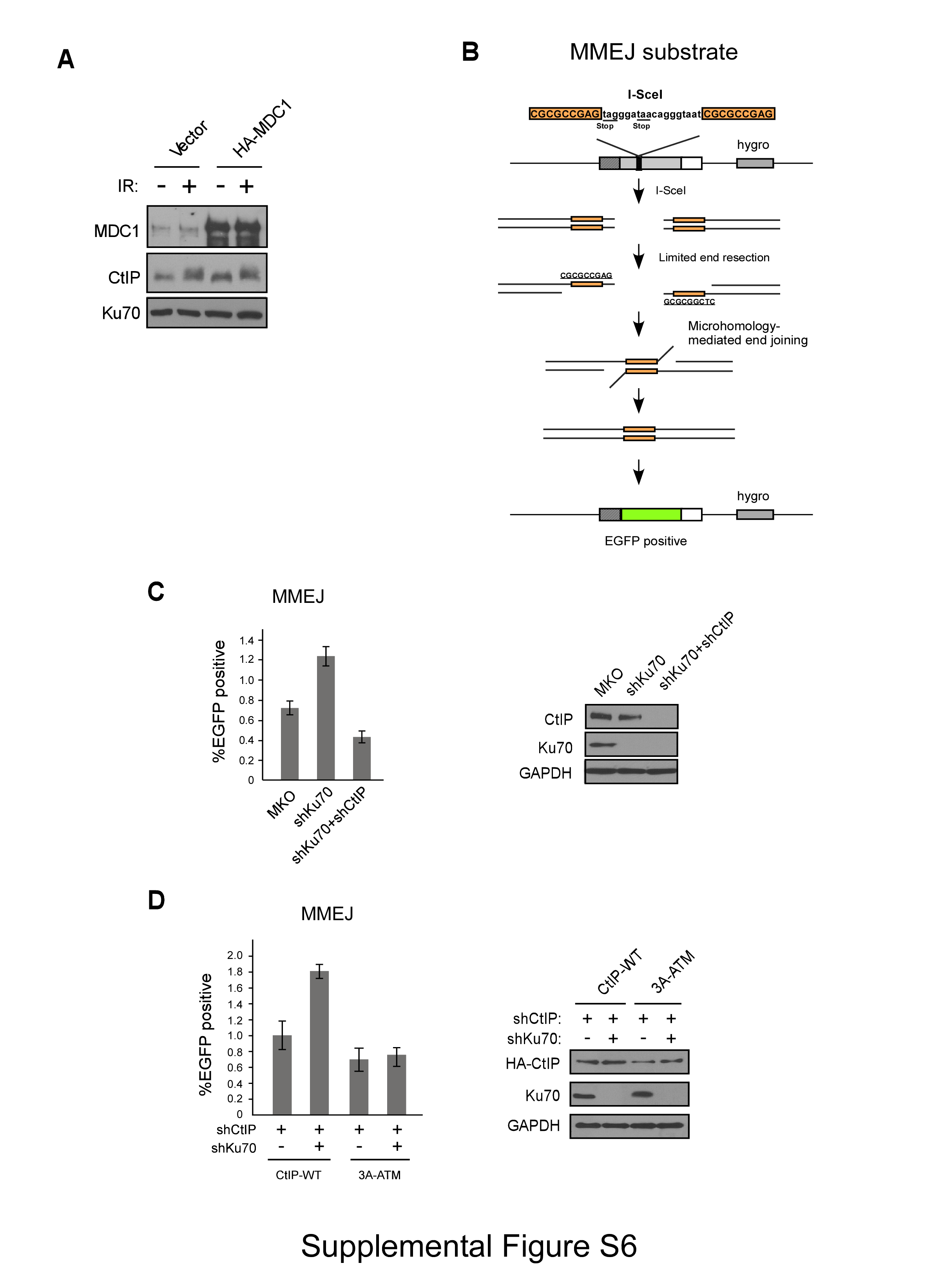

Supplement: Figure S6 — A. Overexpression of MDC1 does not affect CtIP hyper-phosphorylation by ATM. U2OS cells expressing vector control or HA-tagged MDC1 were treated with or without IR (10 Gy, recovered for 1 h), lysed, and immunoblotting was performed with indicated antibodies. B. Schematic drawing of the EGFP-MMEJ repair assay substrate, as previously described [49]. A full-length EGFP cassette was inactivated by inserting a 27-bp oligonucleotide containing an I-SceI cleavage site flanked on both sides by 9-bp microhomology sequence. Upon I-SceI induced generation of DSBs, limited end resection reveals the 9-bp microhomology region needed for annealing and repair of the DSB to generate a functional EGFP cassette. C. EGFP-MMEJ assays were performed with U2OS cells stably expressing control MKO, sh-Ku70 or both sh-Ku70 and sh-CtIP. Western blotting was performed to show silencing of KU70 and CtIP, with GAPDH as a loading control. D. EGFP-MMEJ assays were performed in U2OS cells stably expressing CtIP-WT or 3A-ATM mutant, with endogenous CtIP or both CtIP and Ku70 silenced by shRNAs. Relative repair frequencies were calculated by normalizing the percentage of induced repair to control (CtIP-WT with shCtIP), which is set to 1. Data shown represents the mean of three independent experiments; error bars, s.d. Western blot shows expression of HA-CtIP variants, with GAPDH as a loading control. (TIF) [file pgen.1003277.s006.tif]
