# Supplementary material for: The Interaction of CtIP and Nbs1 Connects CDK and ATM to Regulate HR–Mediated Double-Strand Break Repair
Source: PLoS Genet. 2013 Feb 28;9(2):e1003277. doi: 10.1371/journal.pgen.1003277 (PMC3585124; doi:10.1371/journal.pgen.1003277)
Supplement: Table S1 — Phosphorylated peptides of endogenous CtIP (from HeLa cells) were identified by mass spectrometry. All recovered peptide sequences are listed, with phosphorylated residues labeled with (p). (PDF) [file pgen.1003277.s007.pdf]

Table S1

CtIP SP/TP site	Recovered peptide sequence
S10	G.SSCGS(p)PNSADTSSDFKDLWTK.L
S163	K.IENDQQHQAAELECEEDVIPDS(p)PITAFSFSGVNR.L
	K.IENDQQHQAAELECEEDVIPDS(p)PITAFSFSGVNRL.R
	E.CEEDVIPDS(p)PITAFSFSGVNR.L
	E.CEEDVIPDS(p)PITAFSFSGVNR.L
	E.DVIPDS(p)PITAFSFSGVNR.L
S233	K.VSKSSTHPQHNPNENEILVADTYDQSQS(p)PMAK.A
	K.SSTHPQHNPNENEILVADTYDQS(p)QS(p)PMAK.A
	K.SSTHPQHNPNENEILVADTYDQSQS(p)PMAK.A
	S.STHPQHNPNENEILVADTYDQSQS(p)PMAK.A
	S.STHPQHNPNENEILVADTYDQSQS(p)PMAKAH.G
	S.STHPQHNPNENEILVADTYDQSQS(p)PMAKAH.G
	S.STHPQHNPNENEILVADTYDQSQS(p)PMAKAHGTS.S
	E.ILVADTYDQSQS(p)PMAK.A
	E.ILVADTYDQSQS(p)PMAKAHGTSSYTPDKS.S
T245	K.AHGTSSYT(p)PDKSSFNLATVVAE.T
S276	K.AHGTSSYTPDKSSFNLATVVAETLGLGVQEESETQGPMS(p)PLGDELYHCLEGNHK.K
	K.AHGTSSYT(p)PDKSSFNLATVVAETLGLGVQEESETQGPMS(p)PLGDELYHCLEGNHK.K
	K.AHGTS(p)SYTPDKSSFNLATVVAETLGLGVQEESETQGPMS(p)PLGDELYHCLEGNHK.K
	K.AHGTSSYTPDKSSFNLATVVAETLGLGVQEESETQGPMS(p)PLGDELYHCLEGNHKK.Q
	K.SSFNLATVVAETLGLGVQEESETQGPMS(p)PLGDELYHCLEGNHK.K
	E.TLGLGVQEESETQGPMS(p)PLGDE.L
	E.TLGLGVQEESETQGPMS(p)PLGDELYHCLE.G
	E.TLGLGVQEESETQGPMS(p)PLGDELYHCLEGNHK.K
	G.LGVQEESETQGPMS(p)PLGDELYH.C
	G.LGVQEESETQGPMS(p)PLGDELYHCLE.G
	G.LGVQEESETQGPMS(p)PLGDELYHCLEGNHKK.Q
	G.VQEESETQGPMS(p)PLGDELYHCLE.G
	G.VQEESETQGPMS(p)PLGDELYHCLEGNHK.K
	E.ESETQGPMS(p)PLGDELYHCLE.G
	E.ESETQGPMS(p)PLGDELYHCLEGNHK.K
	E.SETQGPMS(p)PLGDELYHCLE.G
	E.SETQGPMS(p)PLGDELYHCLEGNHK.K
	E.SETQGPMS(p)PLGDELYHCLEGNHKK.Q
	E.TQGPMS(p)PLGDELYHCLE.G
	E.TQGPMS(p)PLGDELYHCLEGNHK.K
T315	R.NTEDSLRFSDS(p)T(p)SKT(p)PPQEELPTR.V
	R.NTEDSLRFSDSTSKT(p)PPQEELPTRVS(p)S(p)PVFGATSSIK.S
	R.FSDSTSKT(p)PPQEELPT.R
	R.FSDSTSKT(p)PPQEELPTR.V
	R.FSDST(p)SKT(p)PPQEELPTR.V
	R.FSDS(p)TSKT(p)PPQE
	R.FSDSTSKT(p)PPQEELPTRV.S
	R.FSDSTSKT(p)PPQEELPTRVSS(p)PVFG.A
	R.FSDSTSKT(p)PPQEELPTRVSS(p)PVFGAT.S
	R.FSDSTSKT(p)PPQEELPTRVSS(p)PVFGATS.S
	R.FSDSTSKT(p)PPQEELPTRVSS(p)PVFGATSSI.K
	R.FSDSTSKT(p)PPQEELPTRVSS(p)PVFGATSSIK.S
	R.FSDSTSKT(p)PPQEELPT(p)RVSSPVFGATSSIK.S
	R.FSDS(p)TSKT(p)PPQEELPTRVS(p)SPVFGATSSIK.S
	R.FSDS(p)TSKT(p)PPQEELPTRVSS(p)PVFGATSSIK.S
	R.FS(p)DSTSKT(p)PPQEELPT(p)RVSSPVFGATSSIK.S
S327	R.NTEDSLRFS(p)DS(p)TSKTPPQEELPTRVSS(p)PVFGATSSIK.S
	R.NTEDSLRFS(p)DST(p)SKTPPQEELPTRVS(p)SPVFGATSSIK.S
	KTPPQEELPT(p)RVSS(p)PVFGATSSIK.S
	R.NTEDSLRFSDSTSKT(p)PPQEELPTRVS(p)S(p)PVFGATSSIK.S
	R.FSDSTSKT(p)PPQEELPTRVSS(p)PVFG.A
	R.FSDS(p)TSKTPPQEELPTRVSS(p)PVFG.A
	R.FSDSTSKT(p)PPQEELPTRVSS(p)PVFGAT.S
	R.FSDSTSKTPPQEELPTRVSS(p)PVFGATS.S
	R.FSDSTSKT(p)PPQEELPTRVSS(p)PVFGATS.S

	R.FSDSTSKT(p)PPQEELPTRVSS(p)PVFGATS.S
	R.FSDSTSKTPPQEELPTRVSS(p)PVFGATSSI.K
	R.FSDSTSKT(p)PPQEELPTRVSS(p)PVFGATSSI.K
	R.FSDSTSKTPPQEELPTRVSS(p)PVFGATSSIK.S
	R.FSDSTSKT(p)PPQEELPTRVSS(p)PVFGATSSIK.S
	R.FSDS(p)TSKT(p)PPQEELPTRVSS(p)PVFGATSSIK.S
	K.TPPQEELPTRVSS(p)PVFGATS.S
	K.TPPQEELPTRVSS(p)PVFGATSSIK.S
	K.TPPQEELPTRVSS(p)PVFGATSSIKSGLDLNTSLSPSLLQPGKK.K
	R.VSS(p)PVFGATSSIK.S
S347	K.SGLDLNTSLS(p)PSLLQPGK.K
	K.SGLDLNTSLS(p)PSLLQPGK.K
	K.SGLDLNTS(p)LS(p)PSLLQPGK.K
	K.SGLDLNTSLS(p)PSLLQPGKK.K
	K.SGLDLNTSLS(p)PSLLQPGKKK.H
S549	K.TIPKGFSSSRKASDGNCTLPKDS(p)PGEPCSQE.C
	K.GFSSSRKAS(p)DGNCTLPKDS(p)PGEPCSQE.C
	R.KASDGNCTLPKDS(p)PGEPCSQE.C
	NCTLPKDS(p)PGEPCSQE.C
	R.KASDGNCTLPKDS(p)PGEPCSQECIILQPLNK.C
	R.KASDGNCTLPKDS(p)PGEPCSQECIILQPLNKCS(p)PDNKPSLQIK.E
	R.KASDGNCTLPKDS(p)PGEPCSQECIILQPLNKCS(p)PDNKPSLQIK.E
	K.ASDGNCTLPKDS(p)PGEPCSQEC
S568	K.DSPGEPCSQECIILQPLNKCS(p)PDNKPSLQ.I
	K.DSPGEPCSQECIILQPLNKCS(p)PDNKPSLQIK.E
	K.DSPGEPCSQECIILQPLNKCS(p)PDNKPSLQIKEENAVFK.I
	K.DSPGEPCSQECIILQPLNKCS(p)PDNKPSLQIKEE.N
	E.CIILQPLNKCS(p)PDNKPSLQIKEENAVFK.I
	N.KCS(p)PDNKPSLQIKEENAVFK.I
S889	R.RQPYNAIFS(p)PK.G
