# Supplementary material for: The Interaction of CtIP and Nbs1 Connects CDK and ATM to Regulate HR–Mediated Double-Strand Break Repair
Source: PLoS Genet. 2013 Feb 28;9(2):e1003277. doi: 10.1371/journal.pgen.1003277 (PMC3585124; doi:10.1371/journal.pgen.1003277)
Supplement: Table S2 — Summary of phosphorylated peptides recovered by mass spectrometry analysis performed using HeLa cells expressing endogenous CtIP and SF9 insect cells expressing recombinant human CtIP (hCtIP). A plus (+) sign indicates that peptides were recovered from indicated, putative SP/TP sites, and a minus (-) sign indicates that peptides were not recovered from indicated site(s). (PDF) [file pgen.1003277.s008.pdf]

Table S2

Phosphorylated SP/TP sites recovered	S10	S163	S233	T245	S276	T315	S327	S347	S549	S568	T847	S889
**HeLa cells Endogenous CtIP**	+	+	+	+	+	+	+	+	+	+	-	+
**Insect cells Recombinant hCtIP**	+	+	+	+	+	+	+	+	+	+	-	+
